# Flush With Data (or) Optimizing and Validating the Efficacy of Free and Computationally Simple 16S Metabarcoding Approaches for Use in Wastewater Surveillance

**DOI:** 10.1111/1462-2920.70276

**Published:** 2026-04-30

**Authors:** Joe Berta, Lori A. Rowe, Evan Multala, Robert F. Garry

**Affiliations:** ^1^ BMS Program Tulane University New Orleans Louisiana USA; ^2^ Tulane University VCIPS Core Covington Louisiana USA; ^3^ Tulane University Neurology New Orleans Louisiana USA; ^4^ Department of Microbiology Tulane University New Orleans Louisiana USA

## Abstract

We propose free and low‐computationally complex methods of 16S rRNA metabarcoding analysis, then optimized and validate their accuracy for wastewater bacterial surveillance. Three taxonomic analysis pipelines were augmented: NCBI BLAST subsampling, Kraken 2/Bracken and QIIME 2/DADA 2. Our optimization strategies for the high complexity of wastewater samples raised QIIME 2/DADA 2's sensitivity to species‐level taxa by 240.5%, while they increased the species‐level selectivity of Kraken 2/Bracken and NCBI BLAST subsampling by 18.7% and 79.1%, respectively. Optimization vastly lowered the read mapping error for BLAST subsampling and Kraken 2/Bracken, by 42.0% and 11.4%, respectively. Microbial community diversity estimates were also improved through our optimization strategies. Richness measurements for BLAST subsampling became 95.6% more accurate, while Kraken 2/Bracken and QIIME 2/DADA 2 improved by 2.2% and 37.8%. Shannon entropy estimates by BLAST subsampling increased in accuracy by 17.4%, while for Kraken 2/Bracken and QIIME 2/DADA 2 they increased by 19.7% and 41.4%. For beta diversity, Bray–Curtis dissimilarity estimates by QIIME 2/DADA 2 increased in accuracy by 8.5% and by Kraken 2/Bracken by 174.3%.

## Graphical abstract

Wastewater treatment plant 16S rRNA sequencing data was used to create simulated read sets of known species composition and relative abundances. These sequence sets were used to optimize current 16S metabarcoding analysis pipelines for the complexity of municipal wastewater samples.

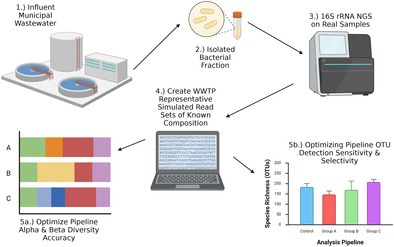

## 1 Introduction

While many advances have recently been made in the surveillance of viral pathogens through the targeted and untargeted analysis of wastewater, surveillance of bacterial pathogens in wastewater has remained largely limited to culture‐based techniques or targeted analysis of select individual species of concern (Carmo dos Santos et al. 2024; Pilevar et al. 2021; Kirsi‐Maarit et al. 2024; Yan et al. 2018; M'ikanatha et al. 2024; Troja et al. 2024). As was evident in our work, many metabarcoding taxonomic classification software can suffer from bias and reproducibility issues when tasked on highly complex bacterial communities, such as are found in municipal wastewater samples (Anastasija et al. 2021; Poussin et al. 2018).

We demonstrate that 16S rRNA amplicon sequencing and metabarcoding analysis can not only serve as a cost‐efficient method of assessing the relative concentration of multiple bacterial pathogens and generate accurate quantification of the bacterial diversity present within a wastewater treatment plant (WWTP), but that it can be done accurately with simple adjustments to accessible taxonomic classification software.

These resulting data can bolster surveillance of human bacterial pathogens and established antibiotic resistance sentinel species. WWTPs rely heavily on bacteria for the processing of sewage and removal of pollutants, and measurements of community composition and bacterial diversity can predict the efficacy of this microbial infrastructure while also creating WWTP‐specific fingerprints for use in sewage contamination tracking in environmental water sources (Zhang et al. 2019, 2020; Siripong and Rittmann 2007). Established data demonstrate that a large proportion of a municipality's wastewater bacteria originate in the urinary and alimentary tracts of its residents, and as such, the WWTP's bacterial community composition can also characterize the catchment population's meta‐microbiome for correlation studies with health markers (Wéry et al. 2010; Newton et al. 2015; Korajkic et al. 2019).

Here we optimized and validated three different free, accessible and widely used taxonomic classification pipelines for application on highly complex bacterial communities: read subsampling with NCBI's BLAST, QIIME 2/DADA 2, and Kraken 2/Bracken (Camacho et al. 2009; Bolyen et al. 2019; Wood et al. 2019). With the decreasing cost of 16S rRNA sequencing now as low as $50–$100 (USD) per sample, it remains one of the most cost‐effective methods of untargeted bacterial detection (Smart et al. 2016; Milián‐García et al. 2021).

Surveillance of enteric bacterial disease incidence is distinctively crucial for many tropical and semi‐tropical low‐income countries, where resources may be limited and bacterial enteric disease burden remains the highest, particularly among children (Kirk et al. 2015; Li et al. 2024; Naghavi et al. 2024). Besides the analysis pipelines being free, all three are suitable for use in environments with fallible infrastructure, with NCBI BLAST subsampling only requiring relatively short duration of reliable internet access and Kraken 2/Bracken and QIIME 2/DADA 2 being capable of fully offline use with locally stored software and reference databases. To further demonstrate the accessibility and low computational complexity of these pipelines, all the work presented within this paper was conducted on rudimentary hardware, specifically using a standard HP Pavilion 15 laptop running Windows 11 with 16GB of RAM and 1 TB of SSD storage.

We determine the accuracy of each pipeline using simulated 16S rRNA sequence sets of known species and abundances made using ART wrapped in CAMISIM (Huang et al. 2011; Fritz et al. 2019). The bacterial composition of these simulated sequencing read sets is composed of species found in samples taken from three different Southeastern Louisiana WWTPs. We evaluated the ability of our analysis pipelines to accurately detect the presence of species‐level taxa, their abundances in the sample, and the alpha (OTUs, Chao1, Pielou's evenness, Shannon entropy) and beta diversity (Bray‐Curtis) of the bacterial community captured in each sample (Chao 1984; Pielou 1966; Shannon 1948; Bray and Curtis 1957). We also propose a novel equation for calculating read mapping error: a weighted, read number‐normalized sum of absolute errors (nSAE) formula. Finally, we show that these optimized pipelines produce results when used on real‐world samples of unknown composition that are consistent with those from our simulated read sets.

## 2 Methods

### 2.1 Sample Collection, Concentration, DNA Extraction, Library Prep and Sequencing

For detailed information regarding collection, processing and sequencing samples and other topics outside the immediate scope of this paper, that is, read QC and standard statistical processes, see in Supporting Information.

### 2.2 Scoring Metrics for Alpha and Beta Diversity, Sensitivity and Selectivity, Read Mapping Error

For detailed information regarding: the calculation of sensitivity, selectivity, read mapping error, alpha and beta diversity, read mapping error modelling, or significance testing, see Scoring Methods in Supporting Information.

### 2.3 Creation of Simulated Read Sets

CAMISIM was used to construct simulated read sets representing each of the three wastewater sampling locations: the West Bank, the North Shore, and New Orleans. In order to choose representative species for each WWTP sampled, each testing location was sampled and sequenced in triplicate. The resulting read sets were then themselves subsampled (*N* = ~1000 merged reads per subsample), and species‐level taxa were taxonomically classified using NCBI's megablast.

The fifteen most dominant species‐level taxa detected at each WWTP, that is, the species with the highest average relative abundance across the three subsamples for each WWTP, were used as representative species in the simulated reads sets. The most abundant species were chosen because we found that dominant taxa were detected by BLAST Subsampling over 2.5× more accuratly than were scarce or low‐density taxa (see NCBI BLAST Subsampling Baseline and Optimized section below). In this way we ensured that the community compostion of our simulated reads included species likely to be encountered in real WWTPs.

Representative nucleotide sequences for simulated read creation were chosen from NCBI based on their relevance in terms of geographical origin and upload date. The five most abundant species, on average, from each WWTP were used as the ‘main taxa’ for that WWTP's simulated read set (see Figure S5). Simulated reads of main taxa were created from three representative nucleotide sequences each, one from each of three relevant strains or subspecies. In the simulated read set, main taxa were given an abundance of 15% each, with each strain/subspecies accounting for 5% of the total abundance of the reads. The second five most abundant species became ‘mid taxa.’ Mid taxa simulated reads were each drawn from the 16S rRNA gene of only one representative strain, accounting per species for 4.5% of the simulated reads. Finally, the last five most abundant species were selected as ‘rare taxa.’ Rare taxa simulated reads were also drawn from a single representative nucleotide sequence but only accounted for 0.5% of the total simulated reads in order to evaluate the pipelines' sensitivity to low‐density taxa.

Within the CAMISIM framework, the ART read simulator was used with parameters set to the 250 bp MiSeq ‘mi’ profile and HiSeq 150 bp ‘hi150’ error profile to create the reads. The mean fragment size was 470 bp with a standard deviation of 12 bp. Read set sizes were set to 0.1 Gb, resulting in ~175 k paired end reads per set.

### 2.4 NCBI BLAST Subsampling Baseline and Optimised Pipelines

(Baseline/Default BLAST Subsampling) Raw forward and reverse fastq.gz sequences were trimmed, paired, and merged using Geneious Prime and its BBDuk and BBMerge plugins (Geneious 2019; Bushnell 2014; Bushnell et al. 2017). One thousand merged reads were subsampled from each sequencing location and fed into NCBI BLASTn and run on Megablast (Chen et al. 2015). Subsamples limited to one thousand reads were used to make the alignment process practical with NCBI's query length restrictions.

(Optimized BLAST Subsampling): In order to optimize mapping selectivity, taxa with fewer reads aligned to them than 0.25 standard deviations less than the mean number of reads distributed among all taxa detected (*z*‐score < −0.25 or reads < μ−0.25*σ*) were excluded from the final analysis. For CAMISIM‐generated read sets, this cutoff number was ~21.4 reads per taxa, while for the more shallowly distributed real read sets, the cutoff was only ~1.5 reads.

This minimum read per taxon threshold, along with the similar threshold used to optimize Kraken 2 results, was added to increase the taxa detection selectivity (see definition in Scoring Methods in Supporting Information) of highly complex samples by decreasing the frequency of Type I errors produced during the read mapping process. This is due to the tendency differences in the distributions of the number of reads mapped to true positive taxa versus those mapped to false positive taxa, as erroneously detected species average far fewer reads mapped to them than those species truly present in the sample (see Figure S6).

When our simulated read sets were taxonomically assigned using NCBI BLAST Subsampling, overall reads were mapped to false‐positive taxa 25.1% of the time. However, taxa with fewer reads than our *z* > −0.25 cutoff had a false‐positive map rate of 79.6%. By removing these low‐read taxa, we increased the average percentage of reads mapped correctly from 76.1% to 85.2%. While a higher cutoff value would theoretically yield even higher read mapping selectivity, we decided to stop at *z* > −0.25 to avoid eliminating the pipeline's ability to detect lower frequency bacteria as much as was practical.

### 2.5 QIIME2 16S CAMISIM Reads Processing

(Baseline/Default QIIME 2/DADA 2) Fastq.gz files containing demultiplexed, untrimmed reads were imported into QIIME2 version 2024.2.0 as ‘Paired End Sequences With Quality’ artefacts. Using the q2‐cutadapt plugin, the reads were then trimmed using the same parameters as described in the Sequencing Methods in Supporting Information. The trimmed reads were then denoised and dereplicated using the q2‐dada2 plugin resulting in a table of amplicon sequence variants (ASVs) and a feature table of each ASV's frequency in the sample (Callahan et al. 2016) (see Figure S7).

These two artefact files were then subject to ‘open reference’ operational taxonomic unit (OTU) clustering with the q2‐vsearch plugin (Rognes et al. 2016; Rideout et al. 2014). Open reference clustering first attempts to collapse the ASVs onto known sequencies from a reference database, as in so‐called ‘closed reference’ OTU clustering. However, in open reference clustering, any remaining ASVs are then clustered ‘de novo,’ where similar leftover ASVs are clustered onto each other to create novel OTUs.

OTUs were clustered at 99% identity to generate species‐level taxonomic data. Three different 16S reference sequence databases were tested which were available at the time of sampling: ‘Greengenes 13_8’ derived from Greengenes 2, as well as ‘Silva 138 SSURef NR99 full‐length sequences’ and ‘Silva 132 SSURef NR99 full‐length sequences’ derived from Silva 132 and 138, respectively (McDonald et al. 2023; Quast et al. 2012). Each was accessed from the QIIME 2 data resources page in February 2024 and imported as FeatureData[Sequence] artefacts. The Silva138 database had exceptionally poor species‐level taxonomic resolution and was removed from consideration.

OTUs were assigned taxonomic classification using the classify‐sklearn plugin of the q2‐feature‐classifier (Robeson et al. 2021; Bokulich et al. 2018; Scikit‐learn 2024). Only classifiers that were already trained and readily available on the QIIME 2 webpage were selected to make the overall pipelines more accessible to researchers with less bioinformatics experience, compared to creating bespoke or uploading and training classifiers. One of four pretrained classifier databases was used during taxonomic assignment, each downloaded from the Qiime2 data resources page in August 2023 (QIIME 2 Data Resources 2024). First is ‘Silva 138 99% OTUs full‐length sequences’ derived from the SILVA SSU database 138. The second was the ‘Greengenes 2 2022.10 full length sequences’ derived from the Greengenes 2 2022 database.

The final two classifiers are variants of the first two, but each trained on 14 different microbial environments, producing classifiers weighted for each species' expected abundance in these populations: ‘Weighted Silva 138 99% OTUs full‐length sequences’ and ‘Weighted Greengenes 13_8 99% OTUs full‐length sequences’. These weighted classifiers were previously found to have increased accuracy in resolving species‐level taxonomy in microbiome samples taken from both distal and proximal animal guts as well as animal secretions, water environments and soil, making them potentially useful for wastewater samples. Greengenes and Silva based taxonomic classifiers were used during OTU clustering in conjunction with their corresponding 16S reference database for ASV creation. An alternate approach of mapping the ASVs directly without clustering to OTUs was also tested, whereas the ASV representative sequences were classified with q2‐feature‐classifier, but no significant difference was observed. Finally, OTU clusters assigned to the same species were combined using qiime2_taxa_collapse.

### 2.6 QIIME 2/DADA 2 Optimised Pipeline Variants

QIIME 2/DADA 2 was tested on simulated reads using a variety of parameter settings. Out of all the reference database/classifier combinations tried, the Greengenes 2 reference database combined with its unweighted classifier was selected for use (see Figure S8).

(QIIME 2/DADA 2 Optimized) Single read set denoising was ‘optimized’ by relaxing the DADA2 parameters to allow for mapping of ‘bimera’ sequences one‐off from the reference sequence, as well as allowing for reads with up to four expected errors instead of the default cap of two (Bash CLI: ‐‐p‐max‐ee‐f 4.0 ‐‐p‐max‐ee‐r 4.0 ‐‐p‐allow‐one‐off True).

(QIIME 2/DADA 2 Double Read Baseline and Optimized) Read sets were also ‘doubled’ by concatenating both the forward and reverse read sets onto themselves before uploading them into the QIIME 2/DADA 2 pipeline. This generated a double read set containing each original read two times, for twice the total reads. These double‐read sets were then themselves run either on default parameters, or optimized with the same loosened DADA2 settings as was used for the optimized single reads but in conjunction with increasing the sci‐kit learn feature classifier's confidence threshold from 0.7 to 0.8 (Bash CLI: ‐‐p‐confidence 0.8).

### 2.7 Kraken 2 16S CAMISIM Reads Processing

(Kraken 2/Bracken 16GB Baseline) FASTQ files containing demultiplexed, trimmed reads were run on Kraken 2 version 2.1.3 as paired reads. The confidence threshold was increased to 0.05 (in arbitrary units) in accordance with one of the author's own suggestions for ‘general purposes’ and other publications, although there is no recommended confidence threshold for Kraken 2, with the default setting being zero (Lu 2021; Wood 2018). Default parameters were used elsewhere (see Figure S9).

Various Kraken suite reference sequence indexes were used in this step; although, as with QIIME 2/DADA 2, only reference indices that were available on the Kraken 2 support page at the time of sampling were used. The Kraken suite provides several different 16S‐specific indexes; however, of them, only Greengenes was tested as it alone provides species‐level resolution. For the whole‐genome Kraken indices, Kraken provides 8 GB and 16 GB condensed versions of the main indices, which were selected for testing here as the complete versions have demanding RAM requirements (between 72 and 171 GBs). The 8 GB and 16 GB versions of the Standard, PlusPF, and PlusPFP indexes were all tested. The best database using the default parameters was the standard Kraken 16 GB database (see Figure S10).

Kraken 2's generated taxonomic report was then fed into Bracken v. 2.9 (Lu et al. 2017, 2022). Species‐level taxonomic reports were generated using 250 bp reads along with default settings and the same reference sequence index as had been used to generate the Kraken 2 report.

### 2.8 Kraken 2/Bracken Optimized Variations

Kraken 2 was run using several different reference databases and strategies.

(Kraken 2/Bracken 16GB Optimized and Kraken 2/Bracken 8 GB Optimized) The two best sensitivity optimization strategies involved using either the 16 GB or 8 GB standard databases with a Kraken 2 confidence threshold of 0.02 and 0.01, respectively. It should be noted that use of any confidence threshold is acceptable in accordance with the guidance given in the Kraken 2 manual, which can be found on their online resources page: github.com/DerrickWood/kraken2/wiki/Manual. Confidence settings of 0.02 and 0.01 were found to perform the best of all confidence settings tested for their respective reference databases.

This was paired with removing all detected taxa that did not have mapped to them at least 0.4% of the total reads to maintain selectivity for CAMISIM read sets. The cutoff threshold used for real read sets was > 0.004% of the total reads due to the more shallow and widely distributed reads found in real samples compared to our simulated read sets of only fifteen taxa. This found the average threshold for simulated reads being ~688 reads mapped per taxa, and ~28 reads mapped per taxa for real read sets. Distribution‐specific *z* value thresholds like those used with BLAST were tested, but due to the irregular read distributions produced by Kraken 2/Bracken, the total read percentage thresholds proved more consistent.

When simulated read sets were taxonomically assigned using our ‘loosened’ or optimized Kraken 2/Bracken parameters with the standard 16GB reference database, reads were only correctly mapped 67.9% of the time, while optimized parameters with the 8GB database resulted in 66.0% accuracy. Taxa with fewer reads than our 0.4% cutoff, meanwhile, were only mapped correctly at a rate of 14.4% for the 16 GB database and 6.7% for the 8 GB database. (see Figure S6) By removing these low‐read taxa, therefore, we increased the average percentage of reads mapped correctly from 67.9% to 71.4% for the 16 GB database and from 66.0% to 77.8% for the 8 GB database.

## 3 Results and Discussion

### 3.1 Part I—CAMISIM Simulated BLAST Subsample, QIIME 2/DADA 2 and Kraken 2/Bracken Compare

#### 3.1.1 Simulated Read Set Accuracy Results

**FIGURE 1:**
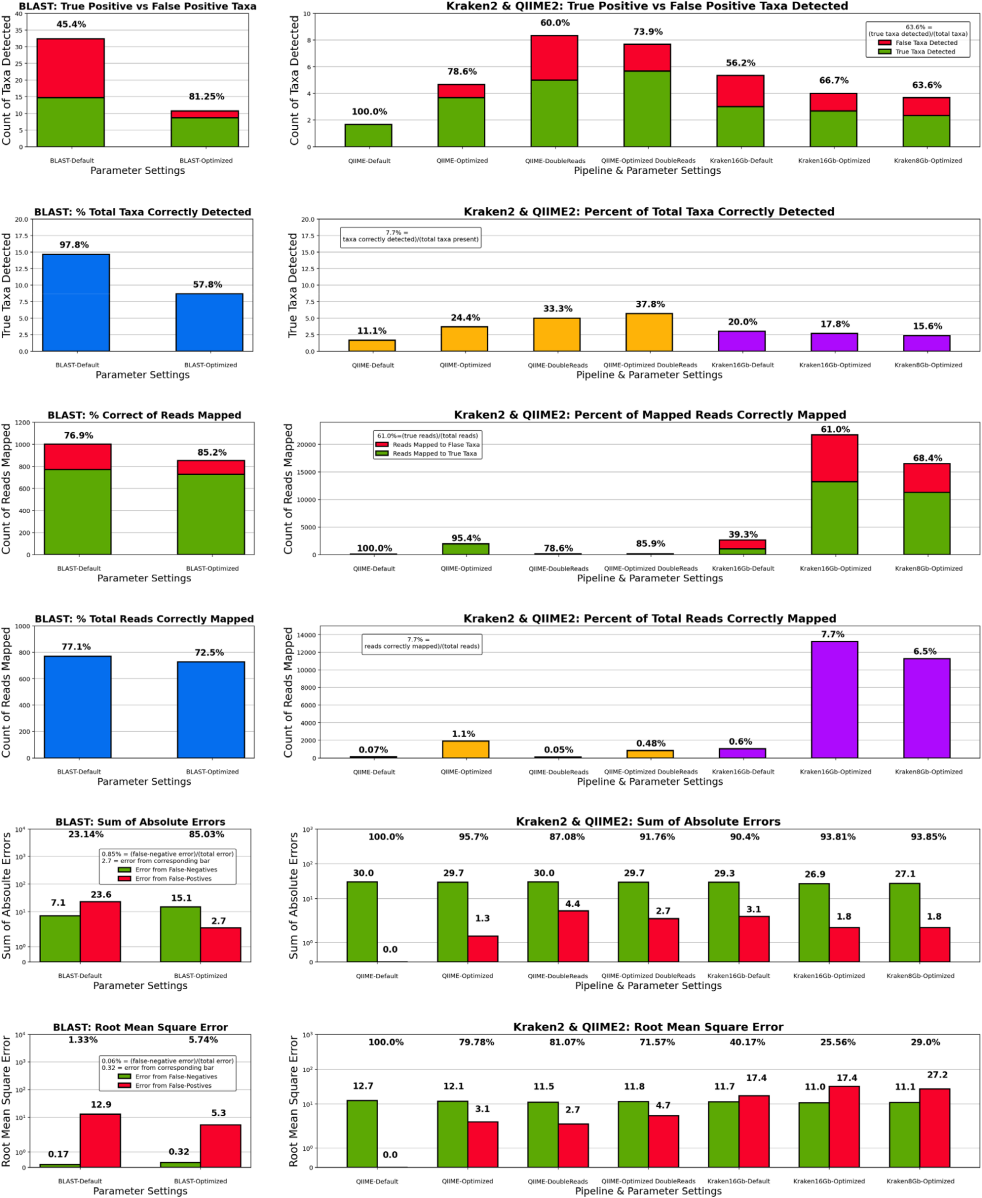
Average taxa detection and read mapping error of different pipelines across the three simulated read sets (top) Total species‐level taxa detected by each pipeline. Taxa correctly detected are given in green and false‐positive taxa are in red to visualise pipeline taxa selectivity (second to top). Out of the total taxa present in the sample, how many were correctly detected by each pipeline. A visual representation of the taxa sensitivity of each pipeline (third from top). Average number of reads mapped by each pipeline. Reads mapped to true taxa are shown in green, while reads mapped to false‐positive taxa are in red to visualise pipeline read mapping selectivity (third from bottom). Out of the total number of reads in the set, how many were successfully mapped to taxa present in the sample. A visualisation of pipeline read mapping sensitivity (second from bottom). Weighted and read‐normalized root mean square error for each pipeline. Error derived from false‐negatives is shown in green, while error from false‐positives is in red (bottom). Weighted and read‐normalized sum of absolute errors for each pipeline. Error derived from false‐negatives is shown in green, while error from false‐positives is in red. Figure created using Matplotlib for Python.

Results for taxa detection and read mapping error are given in Table 1 and Figure 1. Each taxonomic platform started with its own major shortcomings for use on complex microbial samples that needed to be improved: NCBI BLAST Subsampling had near perfect sensitivity but detected more false positives than true positive taxa, QIIME 2/DADA 2 had perfect selectivity but only detected ~10% of the taxa present, and Kraken 2/Bracken had issues with both sensitivity and selectivity, albeit to lesser degrees than either other baseline/default pipeline. These results are indicative of each platform's approach to taxonomy. BLAST is simply returning the ‘best hit’ against the entire NCBI nt database, so nearly all reads are mapped, while QIIME 2/DADA 2 only maps reads that can be collapsed into ASVs, and uses higher confidence thresholds with smaller reference databases. This results in very few reads successfully being mapped, but those that do are very often mapped correctly. In the middle, with features similar to both other pipelines, is Kraken 2/Bracken.

**TABLE 1:** Results of each pipeline for various metrics of read mapping accuracy when used on simulated read sets.

Pipeline	Taxa sensitivity	Taxa selectivity	Read sensitivity	Read selectivity	Mapping error−nSAE	Surveillance score
BLAST‐Default	97.8%	45.4%	77.1%	76.9%	30.7	51.9%
BLAST‐Optimize	57.8%	81.3%	72.5%	85.2%	17.8	54.4%
QIIME‐Default	11.1%	100%	0.07%	100%	30.0	5.6%
QIIME‐Optimize	24.4%	78.6%	1.1%	95.4%	31.0	10.1%
QIIME‐Opt. Double	37.8%	73.9%	0.48%	85.9%	32.4	14.2%
Kraken‐Default	20.0%	56.2%	0.06%	39.3%	32.4	5.7%
Kraken‐Opt. 16 GB	17.8%	66.7%	7.7%	71.4%	28.7	8.4%
Kraken‐Opt. 8 GB	15.6%	63.6%	7.5%	77.8%	28.9	7.6%

*Note:* Values are the average for each pipeline's performance across our three simulated wastewater sequencing read sets. For the definitions of taxa/read sensitivity and selectivity, see Scoring Metrics in Supporting Information. For definitions and modelling of nRMSE and nSAE, see the same.

This made our goals for optimization clear: BLAST Subsampling and Kraken 2/Bracken needed increased selectivity, while Kraken 2/Bracken and QIIME 2/DADA 2 needed increases in sensitivity. To increase sensitivity, we ‘loosened’ parameters in QIIME 2's DADA 2 plug‐in and reduced Kraken 2's confidence threshold from 0.05, the value we used when running with default parameters. This allowed for some read mappings that would have been filtered out by these guardrails to be included, which our simulated read data testing showed more often than not were correctly mapped. Another strategy, discovered completely by accident, was that the doubling of the set of input reads improved QIIME 2/DADA 2's sensitivity dramatically, which we speculate may be due to additional read copies facilitating ASV creation during read denoising. After applying these strategies, taxa sensitivity increases ranged from 30% (Kraken 2/Bracken 16 GB) to 240.5% for QIIME 2/DADA 2.

To increase selectivity, we took advantage of the distribution differences in the number of reads mapped to correct taxa versus those to false positive taxa, specifically their distribution tendencies (see Figure S5). For our baseline BLAST Subsampling and our ‘loosened’ or optimized parameter Kraken 2/Bracken pipelines, false positive taxa tended to have far fewer reads mapped to them than did taxa actually present in the sample. If we assume that each read has a better chance of being mapped correctly than incorrectly, these tendency differences are intuitive. Therefore, we find that for these pipelines there exists some threshold—in reads mapped per taxa—where taxa with fewer reads mapped to them are more likely to be false positive than true positive taxa. Removing these low‐read taxa would preferentially remove false positives. We used a cutoff that was lower than this theoretical threshold to minimise the impact on detection of scarce species truly present in the community, but this still resulted in selectivity increases ranging from 10.9% (Kraken 2/Bracken) to 74.0% (BLAST Subsampling).

### 3.2 Read Mapping Error Equations—RMSE and SAE

As expected from our modelling (see Figure S13), adding weight to the read‐normalized RMSE (nRMSE) derived from false‐negatives made a drastic improvement to the equation's ability to reflect both sources of read mapping error proportionately. Without a weight, nRMSE was negatively correlated (Pearson: *R* = −0.407) with the number of false‐positive taxa, meaning an analysis pipeline that produced more false‐positive taxa paradoxically would have a lower overall error measurement. Adding the weight corrected this to a positive correlation (*R* = 0.691) between false‐positive taxa frequency and total error values.

Regardless, our weighted nSAE formula still outperformed even the weighted nRMSE as a measure of read mapping error. As was apparent from our modelling (see Figures S13–S14, and Table 2), the weighted nRMSE still had a non‐linear relationship between the total taxa detected and mapping error as is typical of root functions, resulting in pipelines that detected relatively few taxa overall incurring more error per false positive or negative taxon detected than did those with higher total taxa counts. While our weighted nRMSE values correlated significantly with both Type II and Type I errors, (Linear regression: type II, 1.36*10^−6^; type I, 3.01*10^−6^) nSAE values correlated more significantly with Type II errors and equally with Type I errors (Linear regression: type II, 9.09*10^−7^; Type I, 2.22*10^−6^). Overall, nSAE proved the more explanatory formula for calculating read mapping errors.

**TABLE 2:** Pearson correlation (*R*) between read mapping error measurement and Type I errors (False Positives) or Type II errors (False Negatives).

Error type	Default RMSE	Read—normalized RMSE	Weighted nRMSE	Default SAE	Read—normalized SAE	Weighted nSAE
R w/Type I errors	−0.509	−0.407	0.691	0.004	0.825	0.698
R w/Type II errors	0.848	0.899	0.708	1.00	0.565	0.716
Average error Corr.	0.170	0.246	0.700	0.502	0.695	0.707

*Note:* Average row reflects the mean correlation with both types that is, how well the measurement reflects overall error.

#### 3.2.1 Simulated Read Set Diversity Results

**FIGURE 2:**
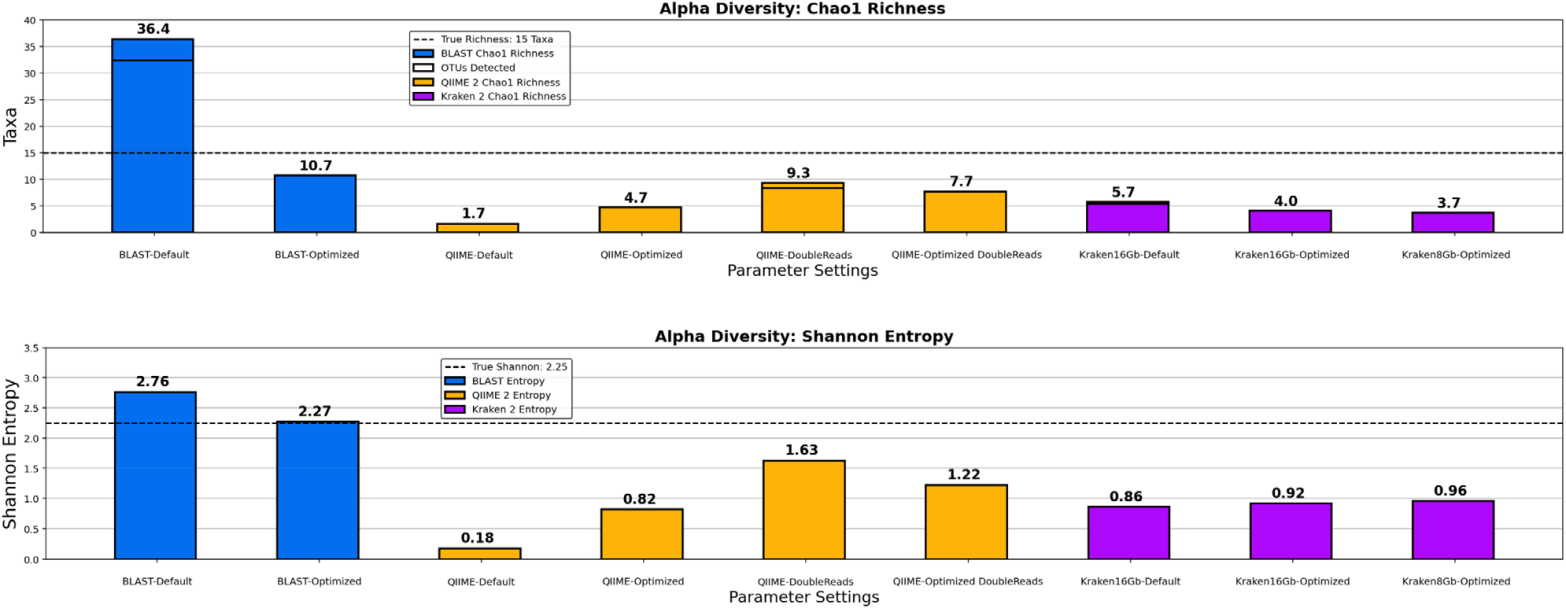
Average alpha diversity detected by different pipelines across the three simulated read sets (top). OTU count and Chao1 predicted richness for each pipeline. Total detected species‐level taxa are marked by a solid black line if different from predicted Chao1 richness. The true richness value of 15 is indicated by dashed line (bottom). Shannon entropy of each pipeline. True entropy of 2.25 is indicated by dashed line. Figure created using Matplotlib for Python.

**FIGURE 3:**
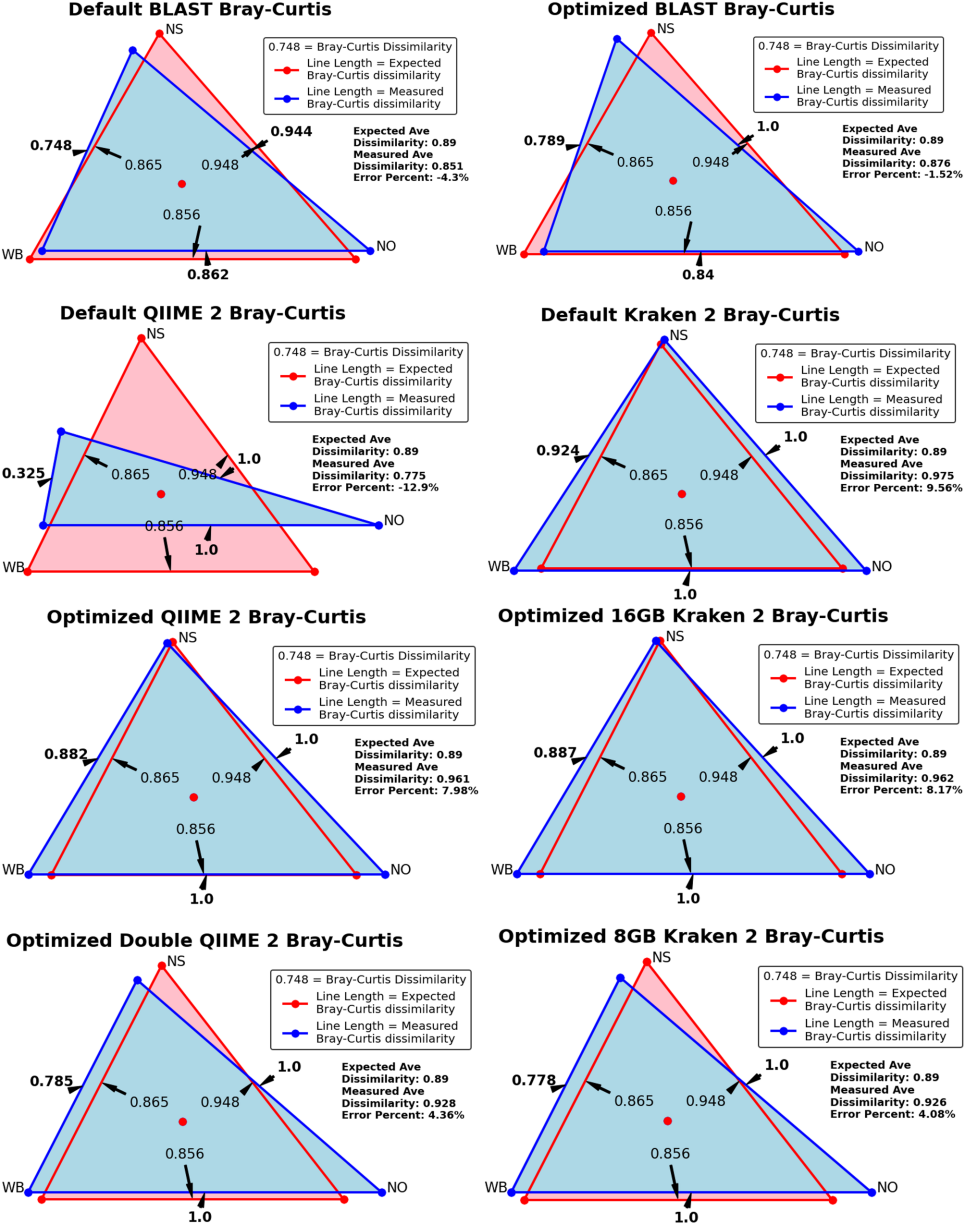
Beta diversity measured by each pipeline between the three simulated read sets. The length of each side corresponds to the Bray–Curtis dissimilarity between two read sets. The true dissimilarity values are given by the red triangle, while the values measured by each pipeline are given in blue. Average dissimilarities are annotated to the right below the legend. Figure created using Matplotlib for Python.

Estimates of alpha and beta diversity from each pipeline are displayed in Figures 2 and 3, respectively. The true value for OTU counts was fifteen taxa per simulated sample, 2.25 bits for Shannon Entropy, 0.83 for Pielou's Evenness index, and the average Bray–Curtis dissimilarity was 0.89. The percent error for each pipeline's estimate from the correct value is given in Table 3. Here our optimisation strategies vastly improved accuracy for nearly every diversity metric tested. While OTU richness estimates remained relatively static with Kraken 2/Bracken, Optimized QIIME 2/DADA 2 accuracy increased by up to 43.6%, and Optimized BLAST Subsampling by up to 82.7%. Shannon entropy error reductions ranged from 24.2% to 78.2%, Bray–Curtis dissimilarity error reductions ranged from 16.3% to 99.9%, and Optimized Kraken 2/Bracken Pielou's evenness error decreased between 40.7% and 57.3% (although it increased for the other pipelines). One unintended consequence is that the benefit of Chao1 richness, which estimates unobserved scarce taxa using the frequency of taxa represented by only one or two reads, was rendered null by our selectivity optimization criteria.

**TABLE 3:** Results of each pipeline for various metrics of alpha and beta diversity when used on simulated read sets.

Pipeline	OTU rich. % error	Chao1 rich. % error	Shannon entropy % error	Pielou even. % error	Bray‐Curtis Diss. % error	Diversity score
BLAST‐Def	+115.6%	+142.4%	+22.5%	−4.2%	−4.3%	63.3
BLAST‐Opt	−20.0%	See OTU	+4.9%	+14.4%	−3.6%	89.3
QIIME‐Def	−86.7%	See OTU	−87.2%	−21.4%	−12.9%	19.7
QIIME‐Opt	−68.9%	See OTU	−63.6%	−25.9%	+8.0%	68.7
QIIME‐Optx2	−48.9%	See OTU	−45.8%	−30.2%	+4.4%	70.3
Kraken‐Def	−64.4%	−62.2%	−66.9%	−15.0%	+174.8%	47.9
Kraken‐Opt16	−64.4%	See OTU	−50.7%	+6.4%	+3.5%	58.4
Kraken‐Opt8	−62.2%	See OTU	−47.2%	+8.9%	+0.5%	67.7

*Note:* Values are the average for each pipeline's performance across our three simulated wastewater sequencing read sets. For the definitions of each diversity metric, see Scoring Methods in Supporting Information.

There was significant variation in the taxa sensitivity depending on the pipeline used, sample location and relative abundance of the taxa (see Figure S16; Table 4). Optimized BLAST Subsampling excelled at detecting dominant taxa. This was followed by QIIME 2/DADA 2, which, when optimized and fed double reads, had decent sensitivity across all abundance levels.

**TABLE 4:** Breakdown of pipeline sensitivity by taxon abundance.

Pipeline	Main taxa sensitivity	Mid taxa sensitivity	Rare taxa sensitivity	False positivity rate
BLAST‐Def	100%	93.3%	100%	54.6%
BLAST‐Opt	100%	73.3%	0%	18.7%
QIIME‐Def	26.7%	6.7%	0%	0%
QIIME‐Opt	46.7%	20.0%	0%	21.4%
QIIME‐Optx2	40.0%	33.3%	20.0%	26.1%
Kraken‐Def	20.0%	20.0%	20.0%	43.8%
Kraken‐Opt16	26.7%	20.0%	6.7%	33.3%
Kraken‐Opt8	20.0%	20.0%	6.7%	36.3%

*Note:* Main taxa are dominant taxa holding 15% abundance each, while mid taxa hold 4.5% abundance, and rare taxa hold only 0.5%. False positivity rate is that rate that the total taxa detected are false positives.

Overall, Kraken 2/Bracken had surprisingly good scores, especially considering that it only detected a single taxon, *Thermomonas carbonis*, from the New Orleans simulated sample, regardless of parameter settings. This is indicative of how the reference database selection can make a critical difference, as follows: follow‐up analysis showed that out of the fifteen taxa chosen to represent the New Orleans WWTP in the simulated read set, *T. carbonis* was, indeed, the only taxon present in the Kraken standard 16GB and 8GB databases, making it the only species able to be detected by our Kraken 2/Bracken pipelines. Omitting the New Orleans sample greatly improved Kraken 2's performance, with the optimized pathways averaging 25% of main taxa, 30% of mid taxa, and 10% of rare taxa.

#### 3.2.2 Relative Abundances

### 3.3 Part II—Real BLAST Subsampling, QIIME 2/DADA 2 and Kraken 2/Bracken Compare

#### 3.3.1 Alpha Diversity

**FIGURE 4:**
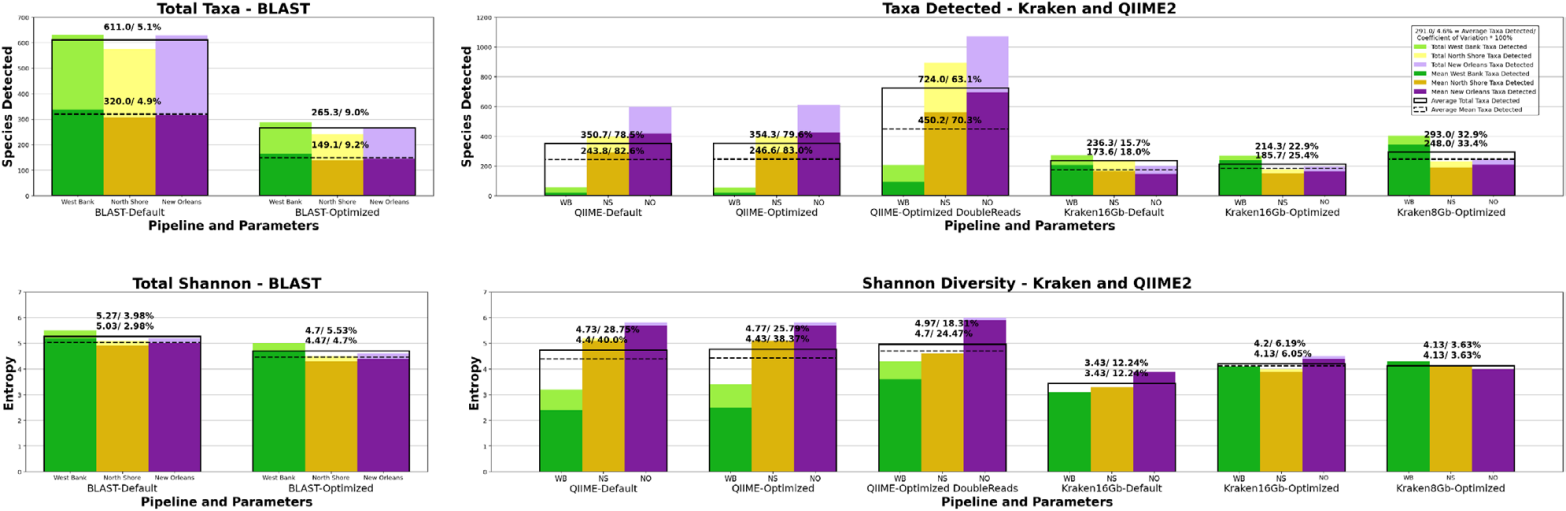
Alpha diversity metrics using real read sets from each of the three sampling locations. Average values corresponding to the three West Bank samples are in green, those for North Shore are gold, while those for New Orleans are purple. Replicate diversity values are shown in dark colors, while site diversity measures are shown in light colors. (See end of method section for definitions) The mean replicate diversity for each metric is represented by dashed black line and the mean site diversity measures are indicated by solid black line. The diversity metrics shown are (top) the total richness in OTUs/species‐level taxa and (bottom) the Shannon Entropy. Figure created using Matplotlib for Python.

When comparing the alpha diversity results of each pipeline derived from our CAMISIM simulated read sets to those of our real read sets, all simulated measures had at least a moderate, positive correlation with results seen on real reads. (Pearson: OTUs, *R* = 0.534; Shannon, *R* = 0.555) (see Table S1). Real samples taken from each WWTP were sequenced in triplicate. Reproducibility can be a common issue in metagenomics, and this replication was done to evaluate precision over multiple measurements. This allowed for two measures to be taken for each alpha diversity metric: the **mean replicate** diversity representing the average alpha diversity measured in each single sequencing replicate across the three sampling sites, while the **mean site** diversity measures represent the total alpha diversity detected in each sampling location across the three sequencing runs done for each site (see Figure 4; Table 5).

**TABLE 5:** Richness metrics of each pipeline when used on real read sets sequenced in triplicate.

Pipeline	OTUs detected per replicate	OTUs detected per site	OTU rep/site	Shannon per replicate	Shannon per site	Mean Bray–Curtis dissimilarity
BLAST‐Def	320.0% ± 4.9%	611.0% ± 5.1%	52.4%	5.03% ± 2.98%	5.27% ± 3.98%	0.718
BLAST‐Opt	149.1% ± 9.2%	265.3% ± 9.0%	56.2%	4.47% ± 4.70%	4.70% ± 5.53%	0.749
QIIME‐Def	243.8% ± 82.6%	350.7% ± 78.5%	69.5%	4.40% ± 40.0%	4.73% ± 28.8%	0.777
QIIME‐Opt	246.6% ± 83.0%	354.3% ± 79.6%	69.3%	4.43% ± 38.4%	4.77% ± 25.8%	0.779
QIIME‐Optx2	450.2% ± 70.3%	724.0% ± 63.1%	62.2%	4.70% ± 24.5%	4.97% ± 18.3%	0.775
Kraken‐Def	173.6% ± 18.0%	236.3% ± 15.7%	73.5%	3.43% ± 12.2%	3.43% ± 12.2%	0.657
Kraken‐Opt16	185.7% ± 25.4%	214.3% ± 22.9%	86.7%	4.13% ± 6.05%	4.20% ± 6.19%	0.646
Kraken‐Opt8	248.0% ± 33.4%	293.0% ± 32.9%	84.6%	4.13% ± 3.63%	4.13% ± 3.63%	0.647

*Note:* Replicate values are the average for each pipeline's performance for each of the nine sequencing replicates taken across our three sampling sites. Site values represent the total diversity detected across the three sequencing replicates taken for each site. The mean of the three sampling sites values is given. For the definitions of diversity metrics, see Figure S4.

The ratio of the mean replicate diversity to the mean site diversity was used to assess each analysis pipeline's precision across multiple sequencing replicates from the same sampling site. Since the sequencing replicates for each site were derived from the same sample, they should theoretically be of similar community composition, barring some variation during aliquoting to create replicates. Mean replicate versus site diversity ratios indicate that BLAST Subsampling became 7.3% more precise, QIIME 2/DADA 2 remained static and Kraken 2/Bracken's precision increased by 18.0%.

#### 3.3.2 Beta Diversity and Relative Abundances

**FIGURE 5:**
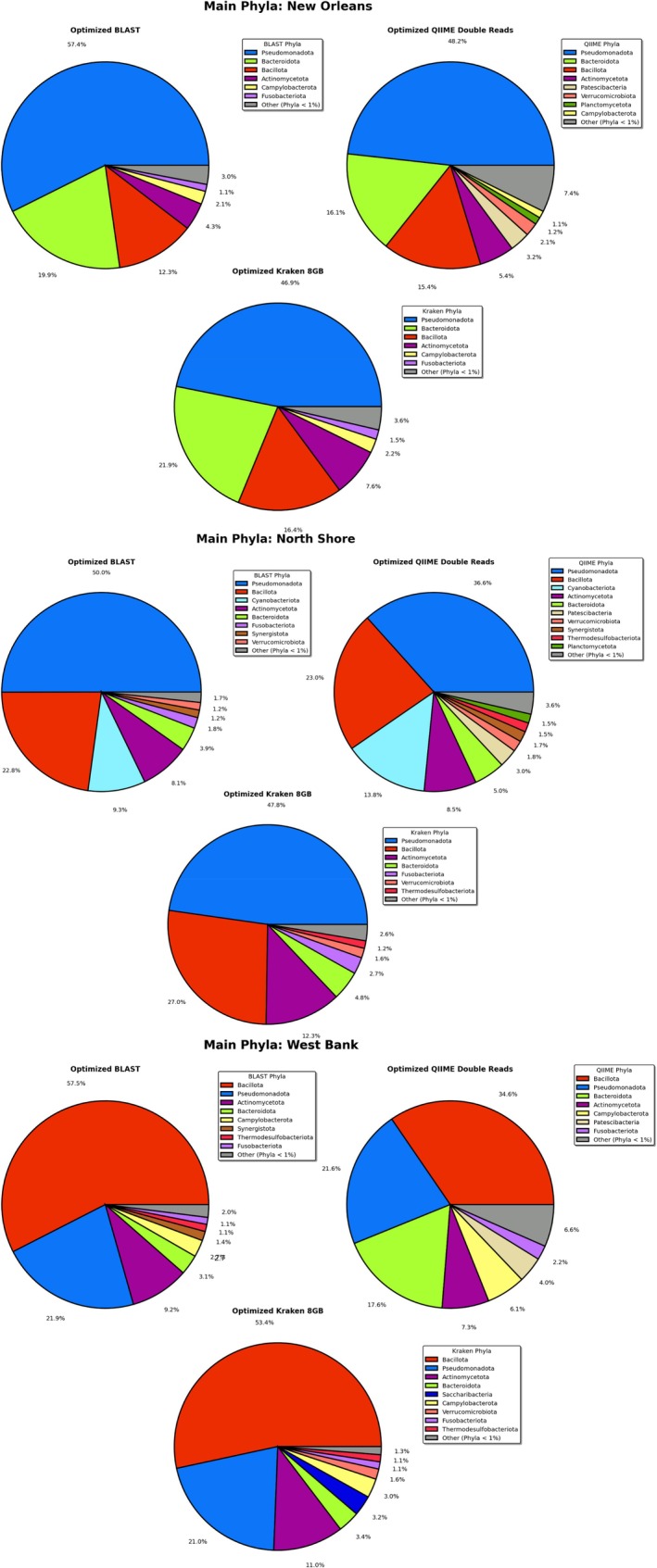
Comparison of the average phyla‐level relative abundance detected by three of the most promising pipelines for each sampling location: (top) the West Bank, (middle) the North Shore, and (bottom) New Orleans. Only phyla that contributed 1% or more of the total relative abundance were shown individually; all other phyla were combined into ‘other’ in grey. Updated phyla nomenclature from Oren A, Garrity GM (2021) was used. Figure created using Matplotlib for Python.

Our West Bank samples were dominated by gram‐positive phyla, the New Orleans sample skewed hard towards gram‐negative phyla, while the North Shore appears to be a rather even mix, only slightly leaning toward Pseudomonadota and the other gram‐negatives (see Figure 5). From this data, it can be inferred that the largest dissimilarity in composition should be observed between the West Bank and New Orleans samples.

**FIGURE 6:**
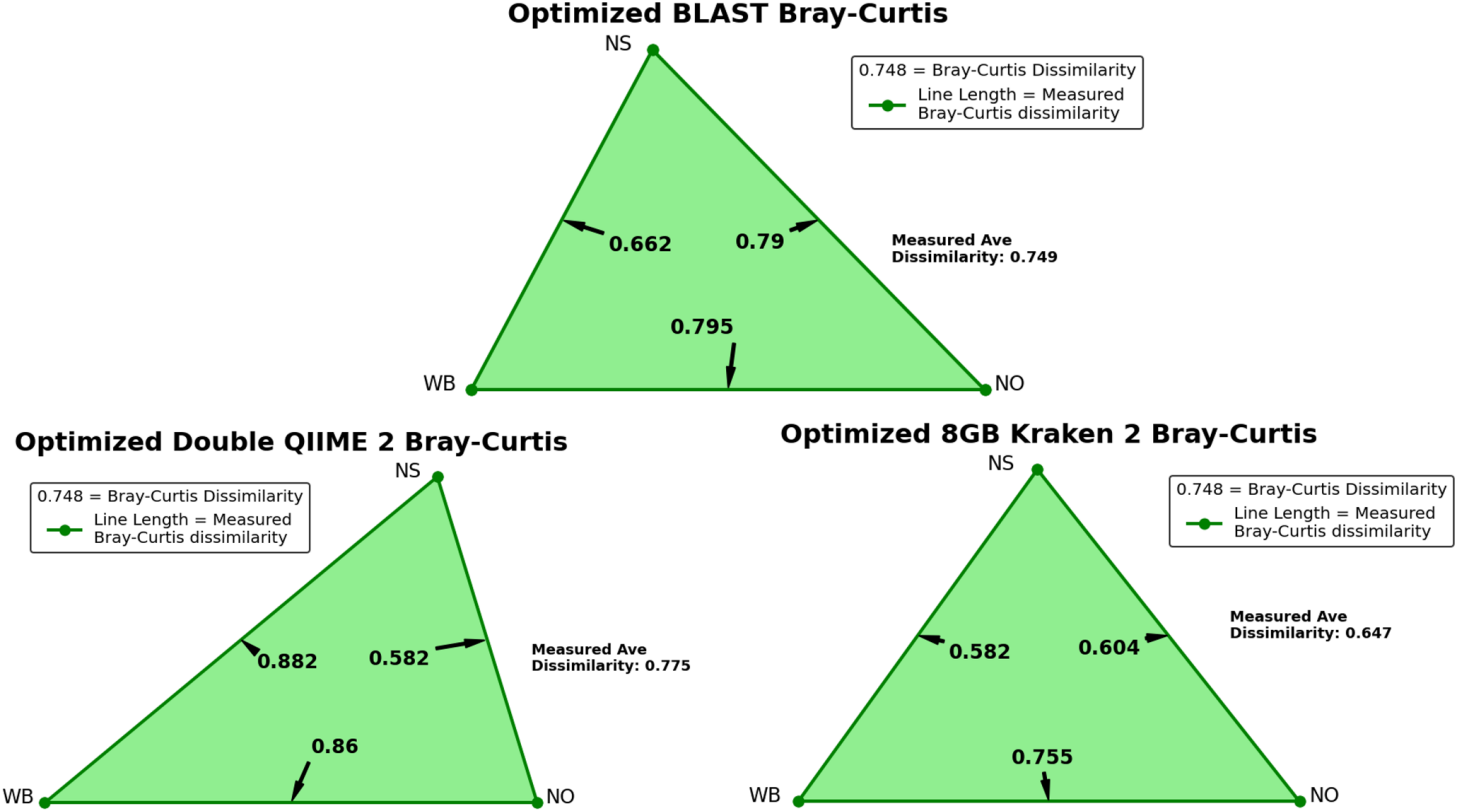
Beta diversity measured by the most promising pipelines using real reads from the three sampling locations. The length of each side corresponds to the average Bray–Curtis dissimilarity detected between the two sites. Average dissimilarities are annotated to the right below the legend. Figure created using Matplotlib for Python.

Out of our three most promising pipelines per our simulated validation: Optimized BLAST Subsampling, Optimized QIIME 2/DADA 2 with double reads, and Optimized Kraken 2/Bracken with the 8GB database, only Optimized QIIME 2/DADA 2 failed to assess the largest Bray–Curtis dissimilarity as being between the West Bank and New Orleans (see Figure 6; Figure S17). It instead detected the most dissimilarity between the West Bank and North Shore populations.

A point of interest is that only QIIME's Greengenes reference database included Patescibacteria, a candidate phyla radiation as of the time of this writing characterised by small cell size and simplified membrane structures (Tian et al. 2020) (see Figure 5). In fact, QIIME 2/DADA 2 tended to overestimate the number and abundance of minor phyla, compared to BLAST Subsampling and Kraken 2/Bracken, while relatively underestimating the abundances of the four major phyla: Pseudomonadota, Bacillota, Actinomycota, and Bacteriodota.

While this may appear errant, it may be another artifact of the minimum read‐per‐taxon optimization strategies used with BLAST Subsampling and Kraken 2/Bracken, and, therefore, QIIME 2/DADA 2 is giving us a more accurate representation of the presence of lower density phyla. This leads to a reasonable inference that optimized QIIME 2/DADA 2 with double input reads was the most sensitive of all the pipelines tested at measuring diversity of real samples at the phyla level.

Unsurprisingly, the dominant phyla in our wastewater samples, Bacteroidota, Bacillota, Actinomycetota, Pseudomonadota, are the same as the dominant phyla in the human gut (Li et al. 2021). Bacteroidota, which are strict anaerobes, may die more rapidly in sewage than Bacillota and Pseudomonadota, which are facultative anaerobes and therefore may engage in aerobic metabolism in oxygenated wastewater.

## 4 Conclusion

In this paper we accomplished multiple objectives. We optimized several popular taxonomic pipelines to be able to be used on the complex microbial communities found in municipal waste with an increased and informed degree of confidence. We propose a novel Sum of Absolute Errors‐based approach to measuring read mapping error and successfully test it against current RMSE‐based models. We succeeded in creating several wastewater taxonomic and diversity analysis pipelines that can be employed by researchers in austere environments or low‐income countries. We also characterised the strengths and weaknesses of each pipeline, allowing investigators to understand which best suits their needs during experimental design.

## Author Contributions

**Joe Berta:** conceptualization, investigation, writing – original draft, writing – review and editing, visualization, validation, methodology, software, formal analysis, data curation. **Evan Multala:** drafting and editing of manuscript, conceptualization. **Lori A. Rowe:** library preperation, sequencing and methodology. **Robert F. Garry:** funding, supervision, logisitics and conceptualization.

## Funding

This work was supported by NIH, 3U01AI151812‐04S1, 561192K2.

## Conflicts of Interest

The authors declare no conflicts of interest.

## Supporting information


**Figure S1:** Map of the Greater New Orleans, LA area, the three WWTPs that were sampled and their approximate catchment areas: Mandeville (North Shore), New Orleans East Bank (NOLA), and Belle Chasse (West Bank). Image created with ArcGIS.
**Figure S2:** The 16S Amplicon Complex using Bakt_341F and Bakt_805R primers to amplify the V3‐V4 region of the 16S rRNA gene of B. adolescentis genome. Figure created with Biorenderer.
**Figure S3:** Alpha rarefaction curves of OTU counts for each pipeline to determine if adequate sequencing depth was achieved with the Illumina V3 and V2 kits. Plateau regions indicate sample sizes of maximum OTU detection. Figures created with vegan for R.
**Figure S4:** Diversity measures used. For alpha diversity: Richness was the number of taxa or OTUs detected, Chao1 was used to estimate the true richness of each sample, Shannon Entropy was used as a measure of diversity (richness and evenness), and Pielou's Evenness as a measure of community evenness. For beta diversity, Bray–Curtis Dissimilarity was used for the differences in two community structures. Singletons/doubletons refer to taxa represented by a single read or two reads, respectively. Figure created using LaTeX.
**Figure S5:** Species present in each our three simulated wastewater 16S read sets: West Bank, North Shore, and New Orleans. Main taxa (15% relative abundance ea.) are shown in bold italics, Mid Taxa (4.5% relative abundance ea.) are shown in underlined italics, and Rare Taxa (0.5% relative abundance ea.) are shown in italics. Figures created with Biorenderer.
**Figure S6:** Violin plot (Box plot in dashed black lines combined with kernel density estimate in colour‐shaded regions). Comparison of the distributions of simulated reads per taxon mapped either correctly (green) or incorrectly (red) for: (A) BLAST Subsampling, (B) Baseline Kraken 2/Bracken 16 GB, (C) Loosened Kraken 2/Bracken 16GB, (D) Loosened Kraken 2/Bracken 8 GB. Figure created with Seaborn for Python.
**Figure S7:** Schematic of the QIIME 2/DADA 2 pipeline. Figure created with Biorenderer.
**Figure S8:** Testing of different QIIME 2/DADA 2 mapping reference and feature classifier combinations at varying mapping confidence settings for taxon detection sensitivity and selectivity using our three simulated read sets. X‐axis displays various confidence thresholds while y‐axis displays correct and incorrect taxa detected. Figure titles indicate reference database and feature classifiers. X‐axis ‘parameter settings’ represent the sci‐kit learn feature classifier confidence threshold with 70% being the default. Figure created using Matplotlib for Python.
**Figure S9:** Schematic of the pipeline used for Kraken 2/Bracken analysis. Figure created using Biorenderer.
**Figure S10:** Testing of different Kraken 2 mapping reference databases at varying mapping confidence settings for taxon sensitivity and selectivity using our three simulated read sets. Figure titles indicate the reference database used. Taxa correctly detected are in green, while false positives are in red. Figure created using Matplotlib for Python.
**Figure S11:** Formulas used to calculate the taxa/read mapping sensitivity and selectivity of the various pipelines and parameters with CAMISIM simulated read sets. Figure created using LaTex.
**Figure S12:** Selection formula for overall read mapping accuracy of our analysis pipelines. Figure created using LaTeX.
**Figure S13:** Modelling of the standard, read‐normalized, and weighted and normalized Root Mean Square Error equations when used to quantify read mapping error for various pipelines on CAMISIM simulated read sets. Each equation was tested using between zero to fifteen different true and false positive taxa detected, increasing by intervals of three. For true taxa detected, each increase of three involved the addition of one main, one mid and one rare taxon detected. Figure created using LaTeX and Matplotlib for Python.
**Figure S14:** Modelling of the standard, read‐normalized, and weighted and normalized Sum of Absolute Errors equations when used to quantify read mapping error for various pipelines on CAMISIM simulated read sets. The number of true and false‐positive taxa was tested the same as with RMSE in Figure S11. Figure created using LaTeX and Matplotlib for Python.
**Figure S15:** Diversity score is calculated with Richness, Pielou's Evenness, Shannon Entropy, and the average value of Bray–Curtis Dissimilarity between the three test sites. The percent error was found for each by comparing the measured and expected value. The mean of these 4% error measures is then subtracted from 100% so the score will vary proportionally with the usefulness of the pipeline for making accurate diversity estimates. Figure created using LaTeX.
**Figure S16:** The relative abundance of each taxon present in each simulated read set for the West Bank, the North Shore and New Orleans. Main taxa, comprising an abundance of 15% each, are shown in shades of green, while mid taxa, comprising 4.5% each, are in shades of blue. Finally, rare taxa, making up only 0.5% each, are shown in shades of red. Grey indicates the relative abundance assigned to false‐positive taxa. Figure created using Matplotlib for Python.
**Figure S17:** Beta diversity measured by the other pipelines for the three real read sampling locations. The length of each side corresponds to the average Bray–Curtis dissimilarity detected between the two read sets. Average dissimilarities are annotated to the right below the legend.
**Table S1:** Split box comparing both simulated and real read results of each taxonomic pipeline. Simulated read results are out of their known true value, while the true values for real reads are unknown. For BLAST Subsampling, Optimized A is with low‐read taxa removed. For Kraken 2/Bracken, Optimized A is optimized parameters with the 16 GB Standard reference database, while Optimized B is the same with the 8GB Standard reference database. For QIIME 2/DADA 2, Optimized A is the optimized pipeline using single‐input reads, while Optimized B is the same for double input reads.

## Data Availability

All raw data is publicly available on https://www.figshare.com/ under project name ‘New Orleans, Mandeville and Belle Chasse, Louisiana Wastewater 16S 09FEB2023’; DOI: 10.6084/m9.figshare.28557443.
