# Supplementary material for: Flush With Data (or) Optimizing and Validating the Efficacy of Free and Computationally Simple 16S Metabarcoding Approaches for Use in Wastewater Surveillance
Source: Environ Microbiol. 2026 Apr 30;28:e70276. doi: 10.1111/1462-2920.70276 (PMC13130369; doi:10.1111/1462-2920.70276)
Supplement: Supplementary file 1 — Figure S1: Map of the Greater New Orleans, LA area, the three WWTPs that were sampled and their approximate catchment areas: Mandeville (North Shore), New Orleans East Bank (NOLA), and Belle Chasse (West Bank). Image created with ArcGIS. Figure S2: The 16S Amplicon Complex using Bakt_341F and Bakt_805R primers to amplify the V3‐V4 region of the 16S rRNA gene of B. adolescentis genome. Figure created with Biorenderer. Figure S3: Alpha rarefaction curves of OTU counts for each pipeline to determine if adequate sequencing depth was achieved with the Illumina V3 and V2 kits. Plateau regions indicate sample sizes of maximum OTU detection. Figures created with vegan for R. Figure S4: Diversity measures used. For alpha diversity: Richness was the number of taxa or OTUs detected, Chao1 was used to estimate the true richness of each sample, Shannon Entropy was used as a measure of diversity (richness and evenness), and Pielou's Evenness as a measure of community evenness. For beta diversity, Bray–Curtis Dissimilarity was used for the differences in two community structures. Singletons/doubletons refer to taxa represented by a single read or two reads, respectively. Figure created using LaTeX. Figure S5: Species present in each our three simulated wastewater 16S read sets: West Bank, North Shore, and New Orleans. Main taxa (15% relative abundance ea.) are shown in bold italics, Mid Taxa (4.5% relative abundance ea.) are shown in underlined italics, and Rare Taxa (0.5% relative abundance ea.) are shown in italics. Figures created with Biorenderer. Figure S6: Violin plot (Box plot in dashed black lines combined with kernel density estimate in colour‐shaded regions). Comparison of the distributions of simulated reads per taxon mapped either correctly (green) or incorrectly (red) for: (A) BLAST Subsampling, (B) Baseline Kraken 2/Bracken 16 GB, (C) Loosened Kraken 2/Bracken 16GB, (D) Loosened Kraken 2/Bracken 8 GB. Figure created with Seaborn for Python. Figure S7: Schematic of th [file EMI-28-e70276-s001.zip › emi70276-sup-0003-Supinfo2.docx]

**Supplementary Sequencing Methods**

Sample Collection

Influent waste from three wastewater treatment plants (WWTPs) were sampled via intake autosampler. At all three locations, one-liter, twenty-four-hour composite samples were collected on Thursday February 9^th^, 2023. The “North Shore” sample was collected from the Mandeville Public Works WWTP in Mandeville, Louisiana, which has a catchment population of around 13,000. **(see Sup. Figure 1)** The “New Orleans” sample was collected from East Bank WWTP located in New Orleans, Louisiana. The New Orleans East Bank has an approximate catchment population of 330,000. The “West Bank” sample was collected from the Plaquemine Parish Water Works in Belle Chasse, Louisiana, which has a catchment population of approximately 10,000.

**Sup. Figure 1** – Map of the Greater New Orleans, LA area, the three WWTPs that were sampled and their approximate catchment areas: Mandeville (North Shore), New Orleans East Bank (NOLA), and Belle Chasse (West Bank). Image created with ArcGIS.


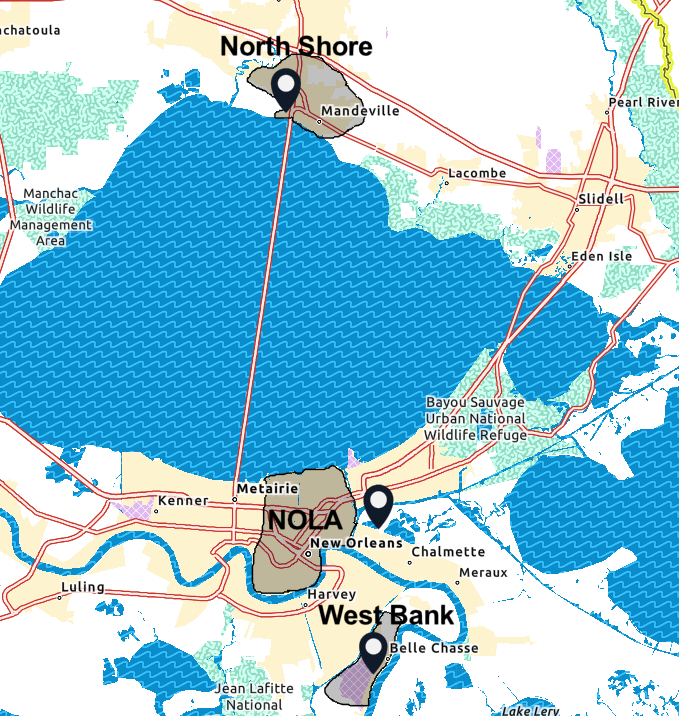
Sample Concentration via High-Speed Centrifugation

One-liter samples were stored at -80°C until ready to be processed. Samples were thawed at 4°C over a 48-hour period then homogenized using a Kaibrite Lab Mixer ran at 2000 RPM for 10 mins per sample. Three 250 mL biological replicates were then subsampled from each one-liter composite sample. After homogenization, 250 mL of sample was pipetted into 250 mL Nalgene centrifuge bottles (Sigma Aldrich B1033) and centrifuged at 500 x g for 5 mins to pellet out large solids. Supernatant was then aliquoted into 4 x 50mL Polypropylene Tube with Snap-On Cap (Beckman Coulter 357005) and centrifuged for 15 mins at RCF_AVE_ 12,000 x g using a JA 25.50 Fixed-Angle Rotor (Beckman-Coulter 363055) to pellet the prokaryotic fraction. Supernatant was pipetted off and prokaryotic pellet was reconstituted in 350 uL 1:1 2X DNA/RNA Shield (Zymo R1100-50) and 2X PBS. Tubes derived from the same subsample were recombined and stored at 4°C overnight.

DNA Extraction

Prokaryotic DNA extraction was conducted according to the Zymo Quick-DNA/RNA Miniprep Plus Kit protocol (Zymo D7003) except for any deviations specified in the following. Briefly, 400 uL samples were homogenized using 0.5 mm BashingBead Lysis Tubes (Zymo S6012-50) ran on Vortex Genie 2 with Microtube Holder (Fischer 50-728-070) set at Speed: 10 for 40 mins. All samples were denatured using a 1:1 volume of DNA/RNA Lysis Buffer (Zymo D7001-1-50), containing guanidinium thiocyanate. DNA was separated from the RNA fraction using Spin-Away Filters (Zymo C1006-50-F) and eluted using 100 uL of nuclease-free water. dsDNA quantity and purity were measured using a Nanodrop OneC Microvolume UV-Vis Spectrophotometer (Fischer 13-400-519). Samples were then refrozen and stored at -20°C until ready to be sequenced. As each sampling location was subsampled in triplicate, these subsample replicates were also extracted separately.


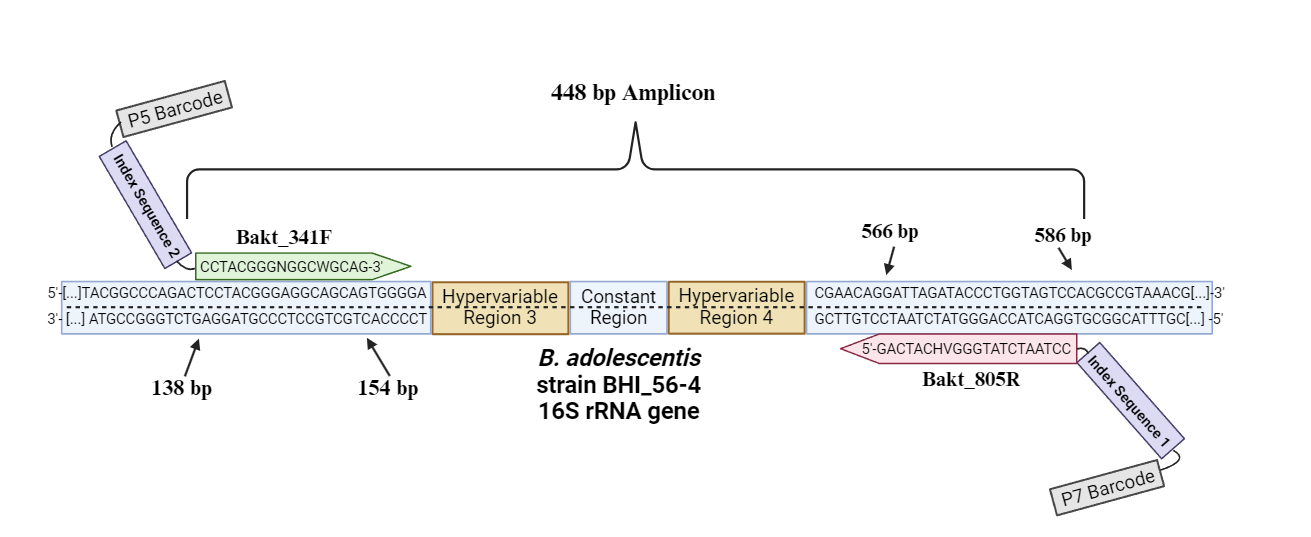
Library Prep and 16S Sequencing

**Sup. Figure 2** – The 16S Amplicon Complex using Bakt_341F and Bakt_805R primers to amplify the V3-V4 region of the 16S rRNA gene of *B. adolescentis* genome. Figure created with Biorenderer.

Library preparation for 16S rRNA sequencing was conducted using the Illumina MiSeq “16S Metagenomic Sequencing Library Preparation” protocol (Illumina, n.d.). This protocol involves two PCR sets, one for the target amplicons and one to add indices and sequence adapters. Both PCR sets involve minimal rounds of PCR to limit sequence-bias introduced by PCR amplification – 25 rounds and 8 rounds, respectively.

The ~400-460 bp amplicons from the V3-V4 regions of the bacterial 16S rRNA gene were created using the Bakt_341F (5’-CCTACGGGNGGCWGCAG-3’) and Bakt_805R (5’- GACTACHVGGGTATCTAATCC -3’) primers originally described by Herlemann et al. in 2011. **(see Sup. Figure 2)** Non-specific forward (5’-TCGTCGGCAGCGTCAGATGTGTATAAGAGACAG-3’) and reverse (5’-GTCTCGTGGGCTCGGAGATGTGTATAAGAGACAG-3’) overhang adapters bridged the 5’ ends of the gene-specific 341/805 primers to the 3’ ends of P5 and P7 barcode sequences using the Nextera XT DNA Library Preparation Kit (Illumina 15032354). 125 ng of each sample was pooled, and the final library pooled concentration was 16.7 ng/µl. The library had a peak height at 630 bp; therefore, the final library was 40.8 nM. This was diluted to 3.82 nM, denatured, and then loaded onto the MiSeq at 10 pM. Cleanup was conducted after each round of PCR using Beckman Coulter’s AMPure XP beads. (Fischer NC9933872)

Each sampling replicate of the three WWTPs were sequenced separately, resulting in three samples per location for a total of nine sets of sequence reads. The sequencing of the West Bank sample was conducted on the Illumina MiSeq system using a V3 Reagent Kit (Illumina) according to the manufacturer’s instructions. Sequencing the 300 x 2 bp library generated an average of 1.6 million reads per sample. North Shore and New Orleans samples were similarly sequenced using a V2 Reagent Kit (Illumina) generating around 623 thousand reads per sample.

This increased sequencing depth for the West Bank samples relative to the rest was intentional. Samples of both relatively deep and shallow sequencing depths were included to determine if trends seen in the abilities of the three different analysis pipelines are robust in the face of such differences, just as WWTPs with both large and small catchment population were included. Since the purpose of this paper is not to compare the diversity of three WWTPs, but to compare and optimize the efficacy of three different analysis pipelines, the West Bank samples did not undergo rarefaction to equalize sequencing depth but were analyzed with ~2x reads as the other samples.

Alpha Rarefaction


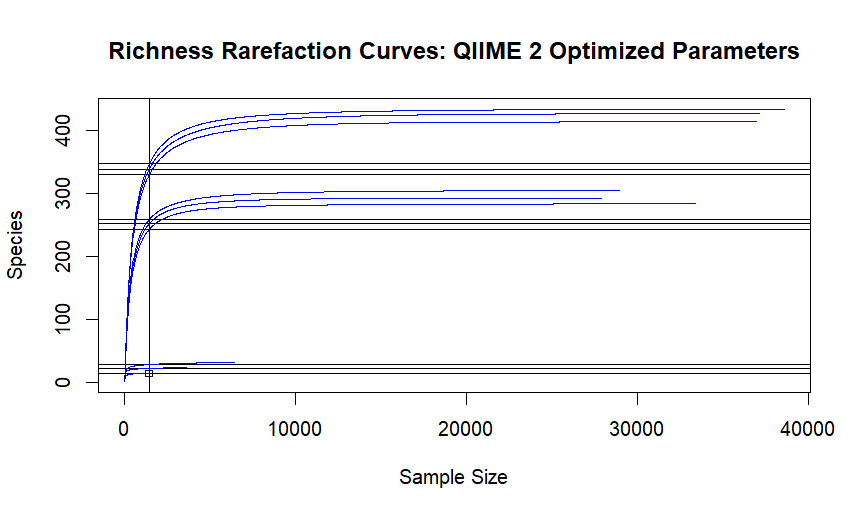

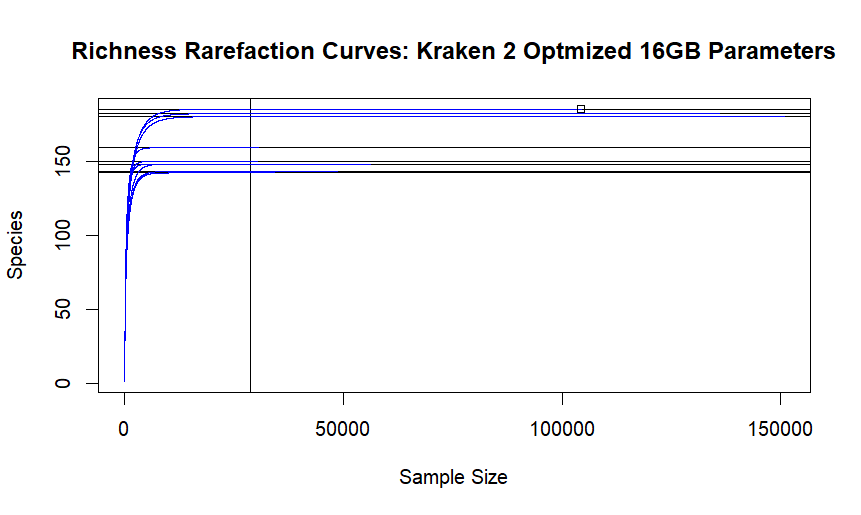

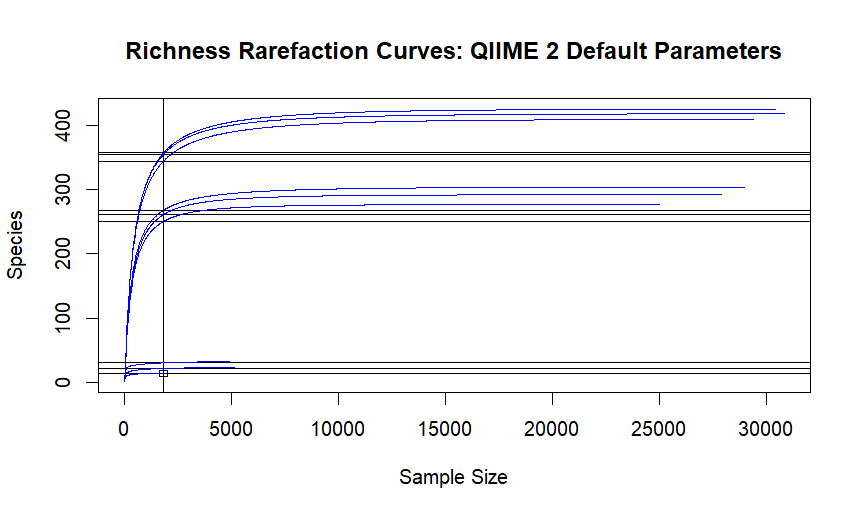

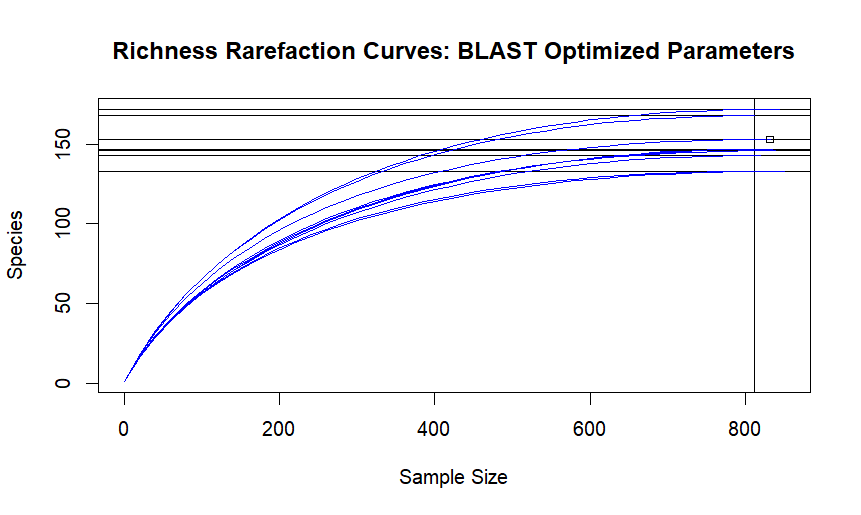

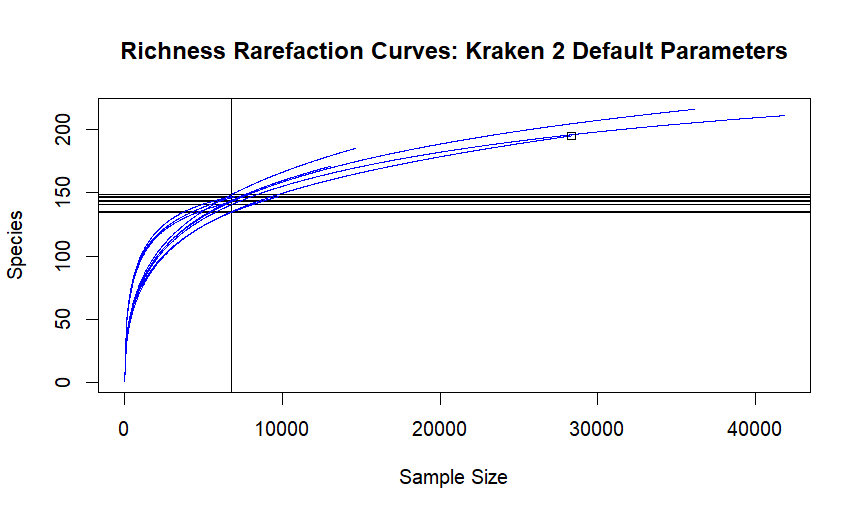

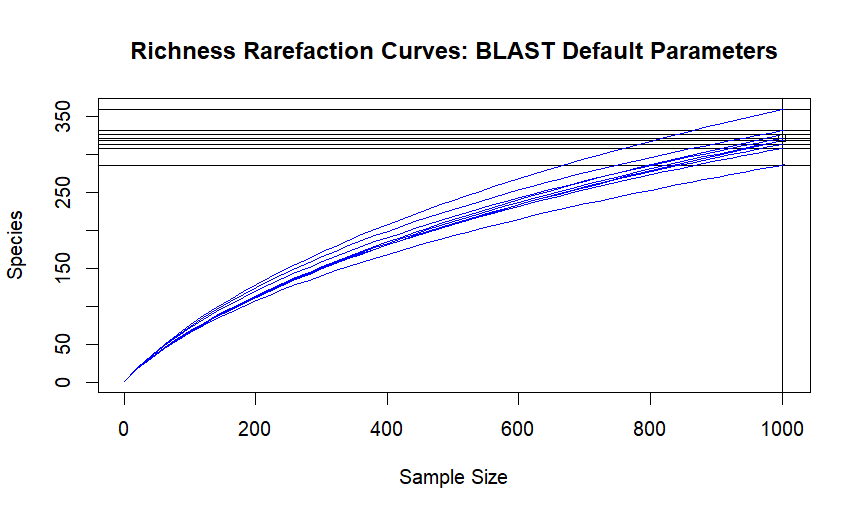
Alpha Rarefaction Curves of OTU counts were utilized to analyze if these read numbers were sufficient for the various pipelines under scrutiny to capture their full potential of species richness. **(see Sup. Figure 3)** These rarefaction curves were created using the Rarefy suite of functions within the VEGAN community ecology package for R (Dixon, 2003). Analysis of these curves indicated that our optimization strategies lowered the necessary sequencing depth for BLAST Subsampling and Kraken 2/Bracken.


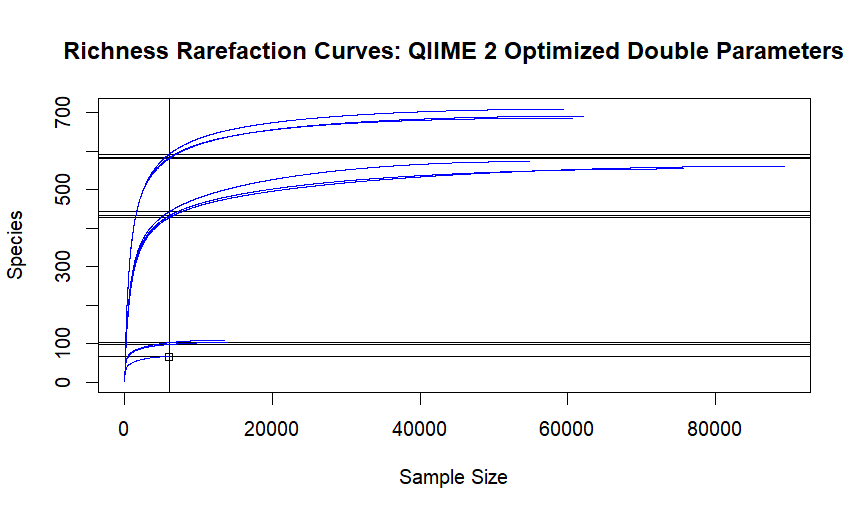

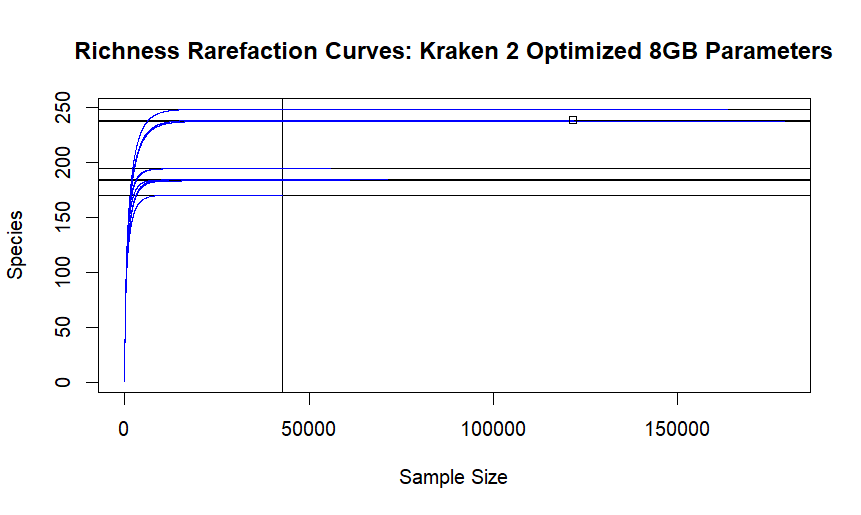


**Sup. Figure 3** – Alpha rarefaction curves of OTU counts for each pipeline to determine if adequate sequencing depth was achieved with the Illumina V3 and V2 kits. Plateau regions indicate sample sizes of maximum OTU detection. Figures created with vegan for R.

Read Quality Control and Trimming

Initial quality control checks for raw reads were conducted using FastQC (Andrews, 2010). Adapter sequences, low-quality sequences and short reads were trimmed using Cutadapt version 4.8 (Martin, 2011). Nextera barcodes, overhang adapters and primer sequencies were removed by targeting of the forward and reverse primers. Bases with a Phred score below Q20 were removed from either end, as were reads truncated below 230 bp to ensure sufficient paired read overlap for merging. This resulted in an average of 918 thousand trimmed reads for the West Bank samples and 580 thousand for New Orleans and the North Shore.

**References**

1. Andrews, S. (2010). Babraham Bioinformatics - FastQC a quality control tool for high throughput sequence data. Babraham.ac.uk. https://www.bioinformatics.babraham.ac.uk/projects/fastqc/
2. Dixon, P. (2003). VEGAN, a package of R functions for community ecology. Journal of Vegetation Science, 14(6), 927–930. <https://doi.org/10.1111/j.1654-1103.2003.tb02228.x>
3. Herlemann, D. P., Labrenz, M., Jürgens, K., Bertilsson, S., Waniek, J. J., & Andersson, A. F. (2011). Transitions in bacterial communities along the 2000 km salinity gradient of the Baltic Sea. The ISME Journal, 5(10), 1571–1579. https://doi.org/10.1038/ismej.2011.41
4. Illumina 16S Metagenomic Sequencing Library Preparation. (n.d.). https://support.illumina.com/documents/documentation/chemistry_documentation/16s/16s-metagenomic-library-prep-guide-15044223-b.pdf
5. Martin, M. (2011). Cutadapt removes adapter sequences from high-throughput sequencing reads. EMBnet.journal, 17(1), 10. https://doi.org/10.14806/ej.17.1.200
