# Supplementary material for: Flush With Data (or) Optimizing and Validating the Efficacy of Free and Computationally Simple 16S Metabarcoding Approaches for Use in Wastewater Surveillance
Source: Environ Microbiol. 2026 Apr 30;28:e70276. doi: 10.1111/1462-2920.70276 (PMC13130369; doi:10.1111/1462-2920.70276)
Supplement: Supplementary file 1 — Figure S1: Map of the Greater New Orleans, LA area, the three WWTPs that were sampled and their approximate catchment areas: Mandeville (North Shore), New Orleans East Bank (NOLA), and Belle Chasse (West Bank). Image created with ArcGIS. Figure S2: The 16S Amplicon Complex using Bakt_341F and Bakt_805R primers to amplify the V3‐V4 region of the 16S rRNA gene of B. adolescentis genome. Figure created with Biorenderer. Figure S3: Alpha rarefaction curves of OTU counts for each pipeline to determine if adequate sequencing depth was achieved with the Illumina V3 and V2 kits. Plateau regions indicate sample sizes of maximum OTU detection. Figures created with vegan for R. Figure S4: Diversity measures used. For alpha diversity: Richness was the number of taxa or OTUs detected, Chao1 was used to estimate the true richness of each sample, Shannon Entropy was used as a measure of diversity (richness and evenness), and Pielou's Evenness as a measure of community evenness. For beta diversity, Bray–Curtis Dissimilarity was used for the differences in two community structures. Singletons/doubletons refer to taxa represented by a single read or two reads, respectively. Figure created using LaTeX. Figure S5: Species present in each our three simulated wastewater 16S read sets: West Bank, North Shore, and New Orleans. Main taxa (15% relative abundance ea.) are shown in bold italics, Mid Taxa (4.5% relative abundance ea.) are shown in underlined italics, and Rare Taxa (0.5% relative abundance ea.) are shown in italics. Figures created with Biorenderer. Figure S6: Violin plot (Box plot in dashed black lines combined with kernel density estimate in colour‐shaded regions). Comparison of the distributions of simulated reads per taxon mapped either correctly (green) or incorrectly (red) for: (A) BLAST Subsampling, (B) Baseline Kraken 2/Bracken 16 GB, (C) Loosened Kraken 2/Bracken 16GB, (D) Loosened Kraken 2/Bracken 8 GB. Figure created with Seaborn for Python. Figure S7: Schematic of th [file EMI-28-e70276-s001.zip › emi70276-sup-0001-Figures.docx]

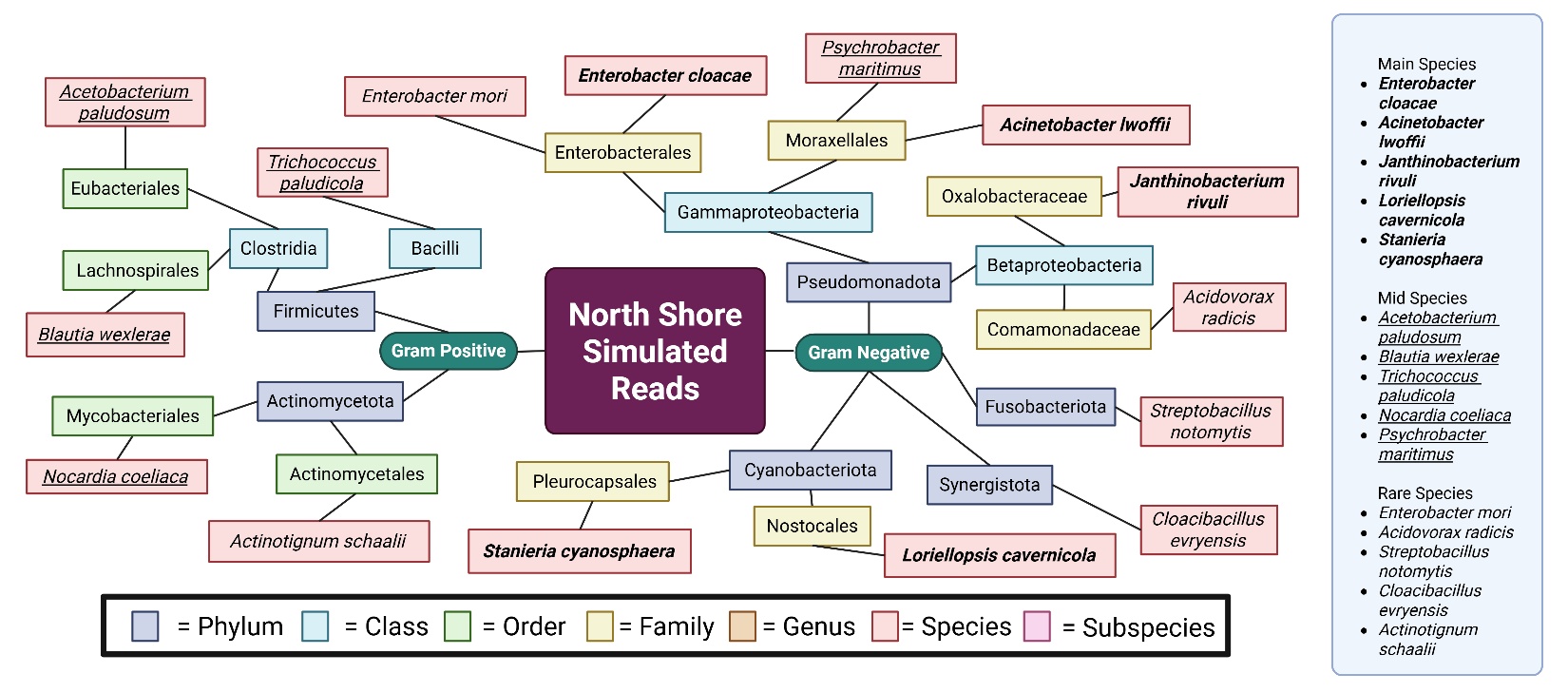

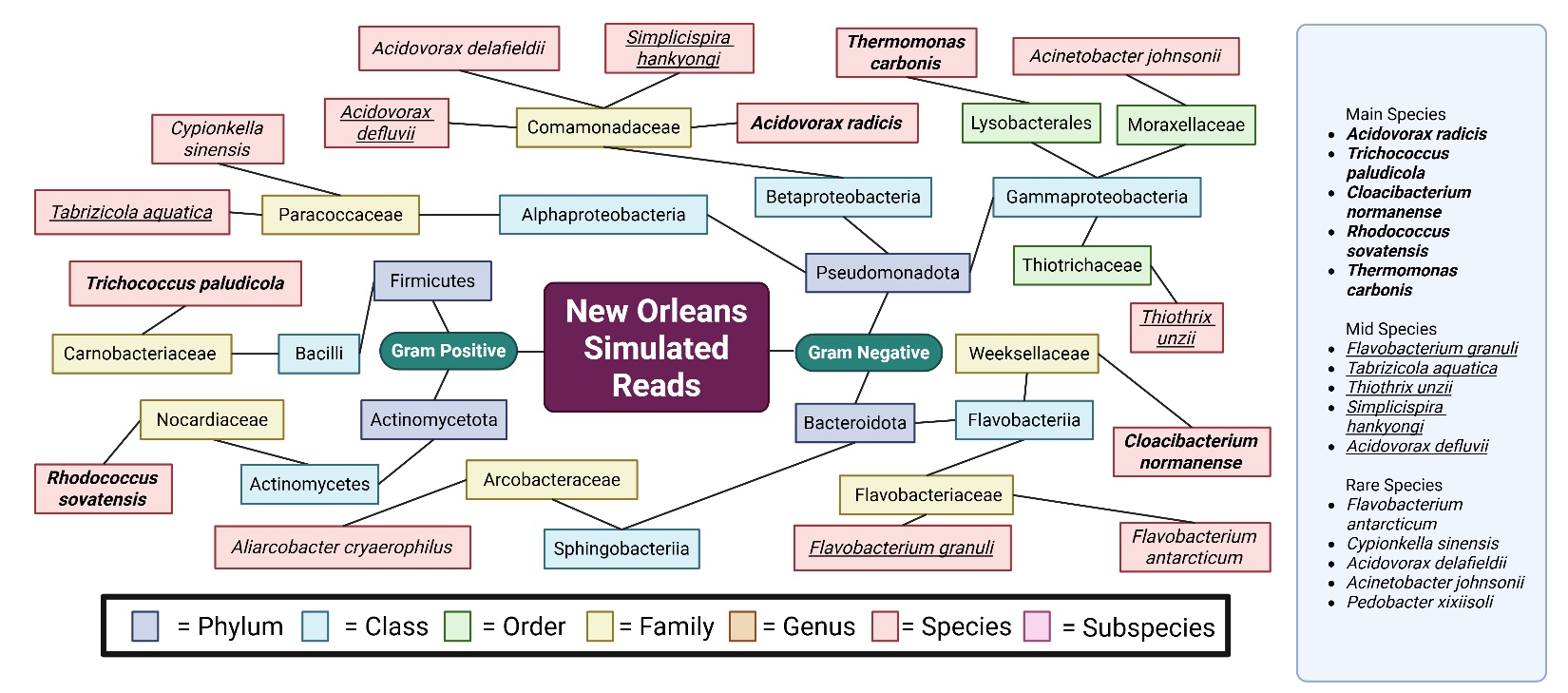


**Sup. Figure 5** – Species present in each our three simulated wastewater 16S read sets: West Bank, North Shore, and New Orleans. Main taxa (15% relative abundance ea.) are shown in bold italics, Mid Taxa (4.5% relative abundance ea.) are shown in underlined italics, and Rare Taxa (0.5% relative abundance ea.) are shown in italics. Figures created with Biorenderer.


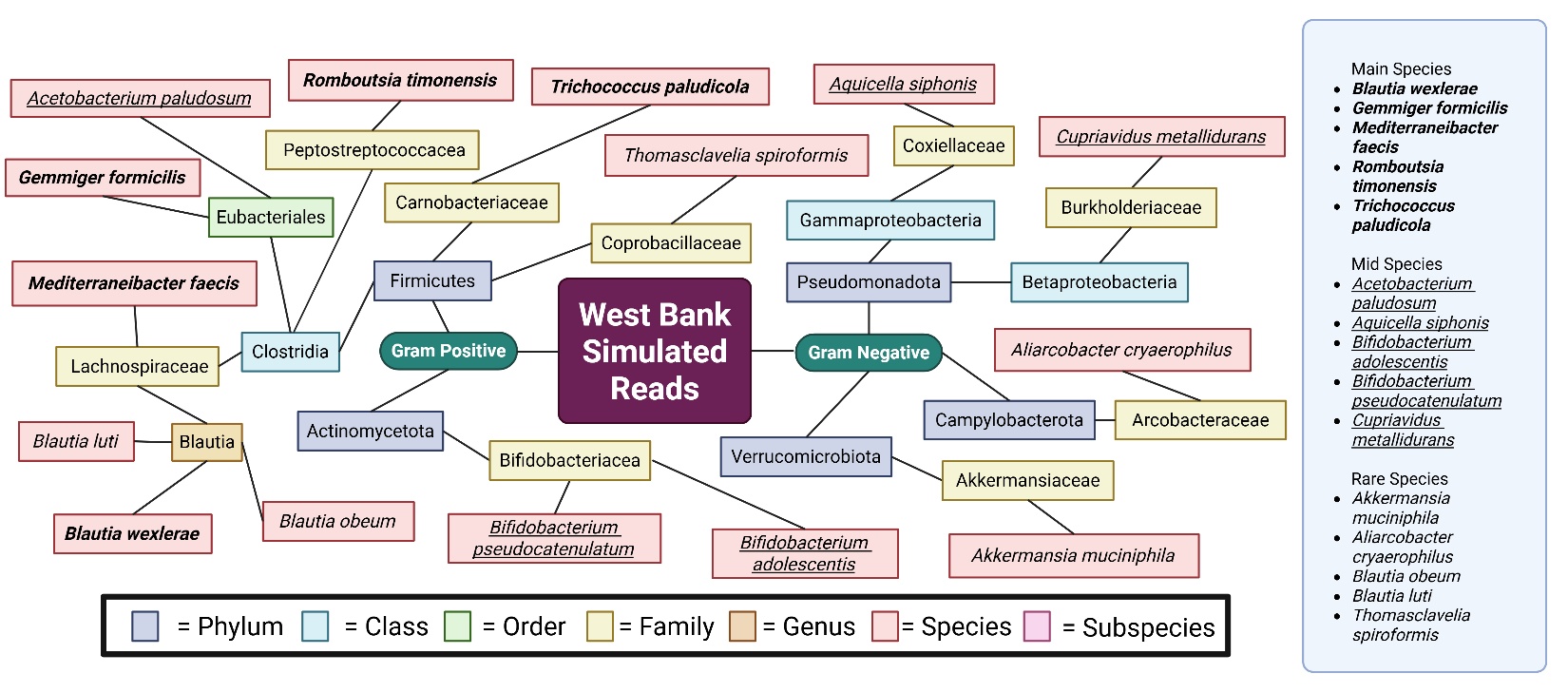


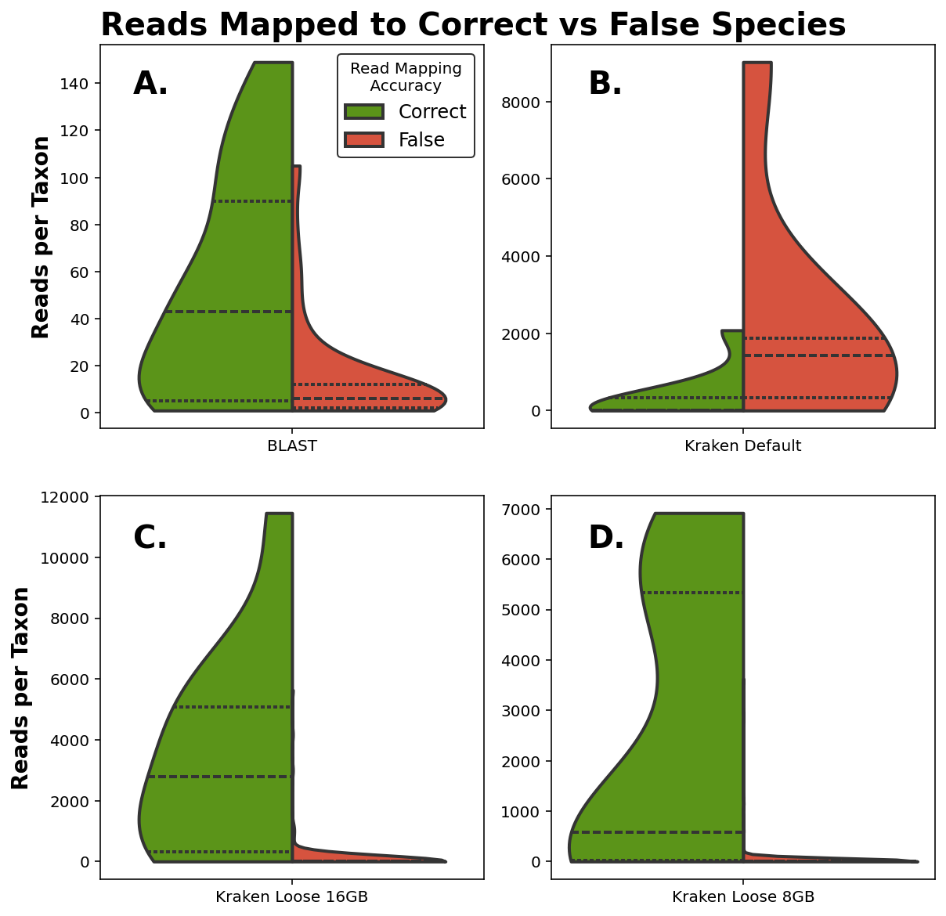


**Sup. Figure 6** – Violin plot (Box plot in dashed black lines combined with kernel density estimate in color-shaded regions). Comparison of the distributions of simulated reads per taxon mapped either correctly (green) or incorrectly (red) for: a.) BLAST Subsampling, b.) Baseline Kraken 2/Bracken 16GB, c.) Loosened Kraken 2/Bracken 16GB, d.) Loosened Kraken 2/Bracken 8GB. Figure created with Seaborn for Python.


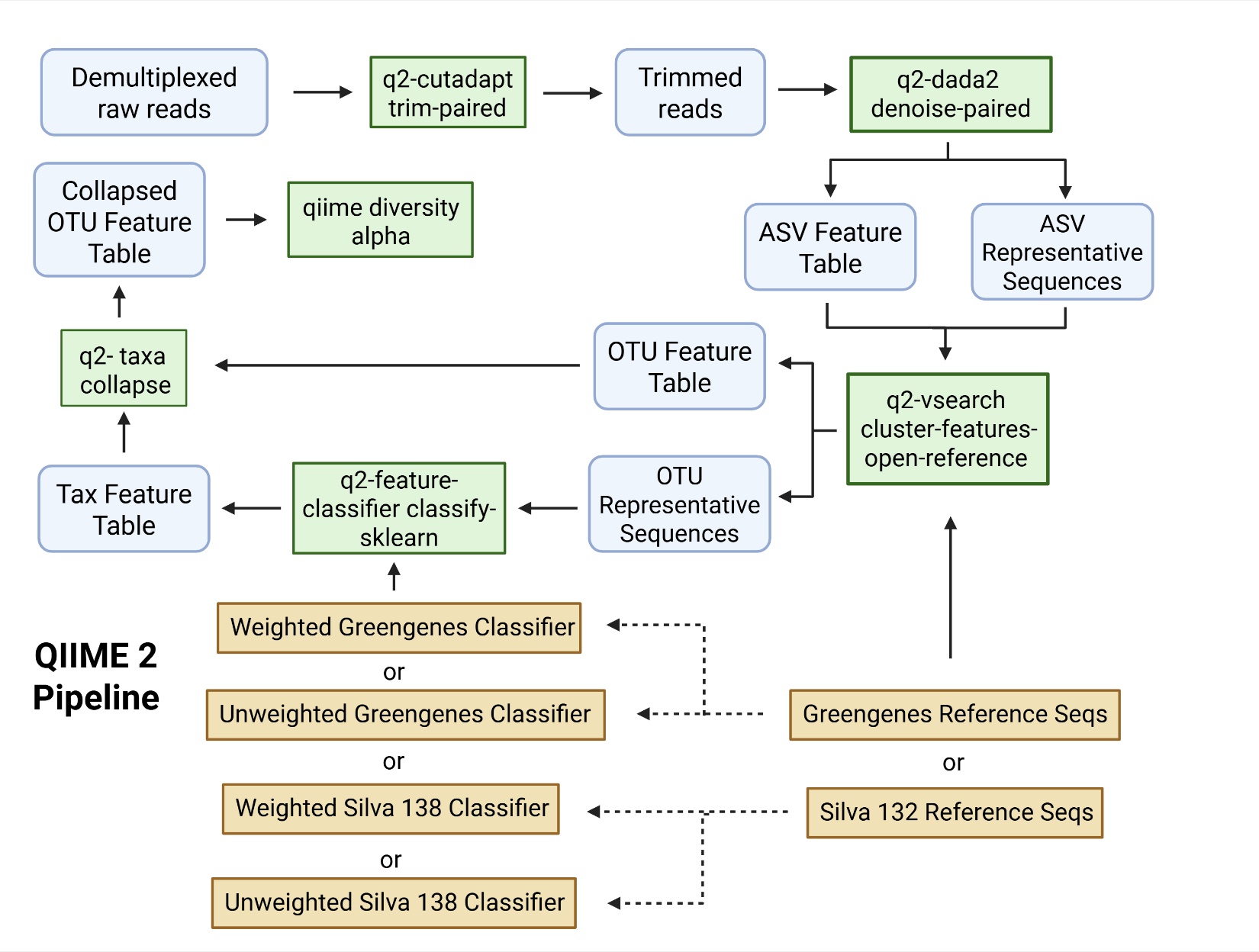


**Sup. Figure 7** – Schematic of the QIIME 2/DADA 2 pipeline. Figure created with Biorenderer.

**Sup. Figure 8** – Testing of different QIIME 2/DADA 2 mapping reference and feature classifier combinations at varying mapping confidence settings for taxon detection sensitivity and selectivity using our three simulated read sets. X-axis displays various confidence thresholds while y-axis displays correct and incorrect taxa detected. Figure titles indicate reference database and feature classifiers. X-axis “parameter settings” represent the sci-kit learn feature classifier confidence threshold with 70% being the default. Figure created using Matplotlib for Python.


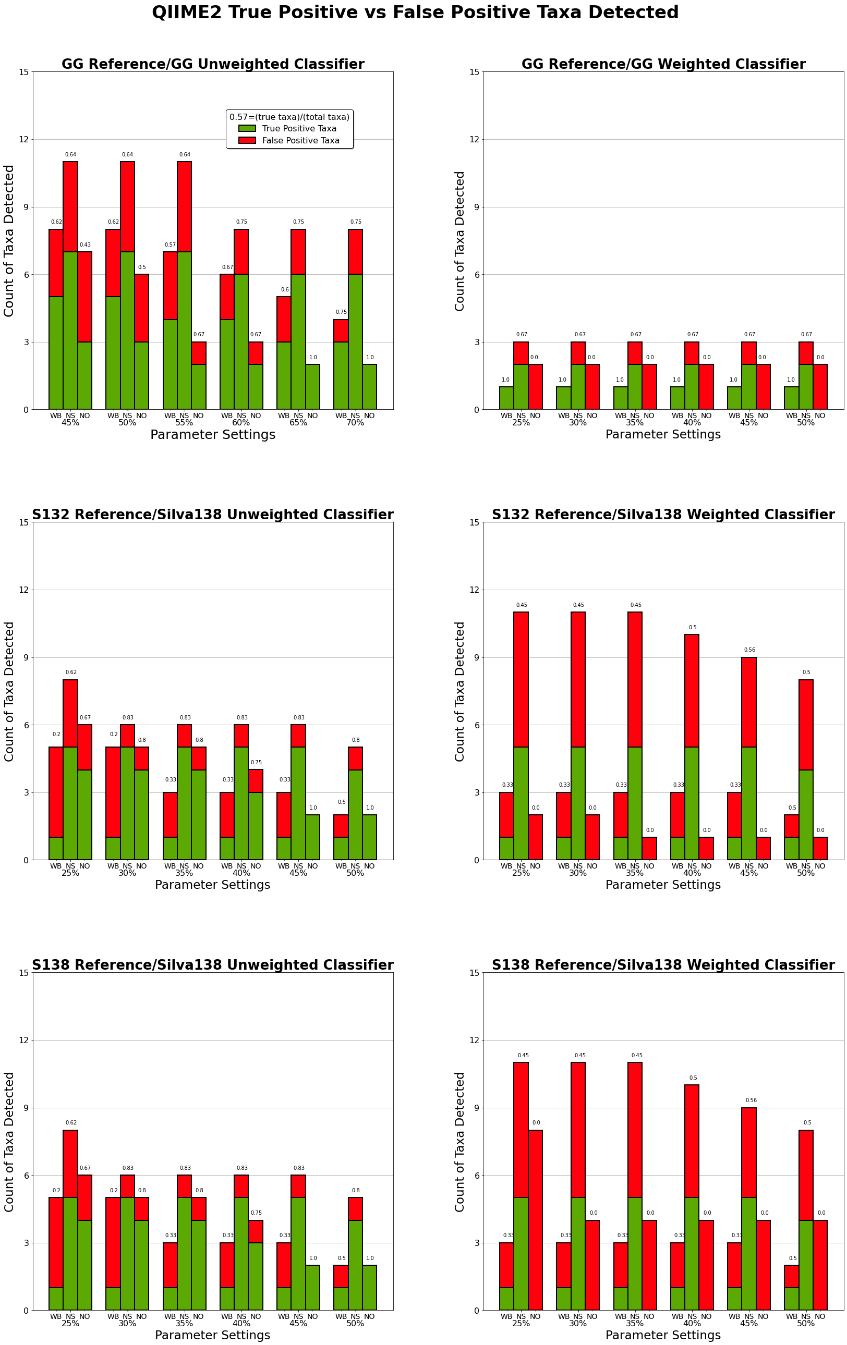


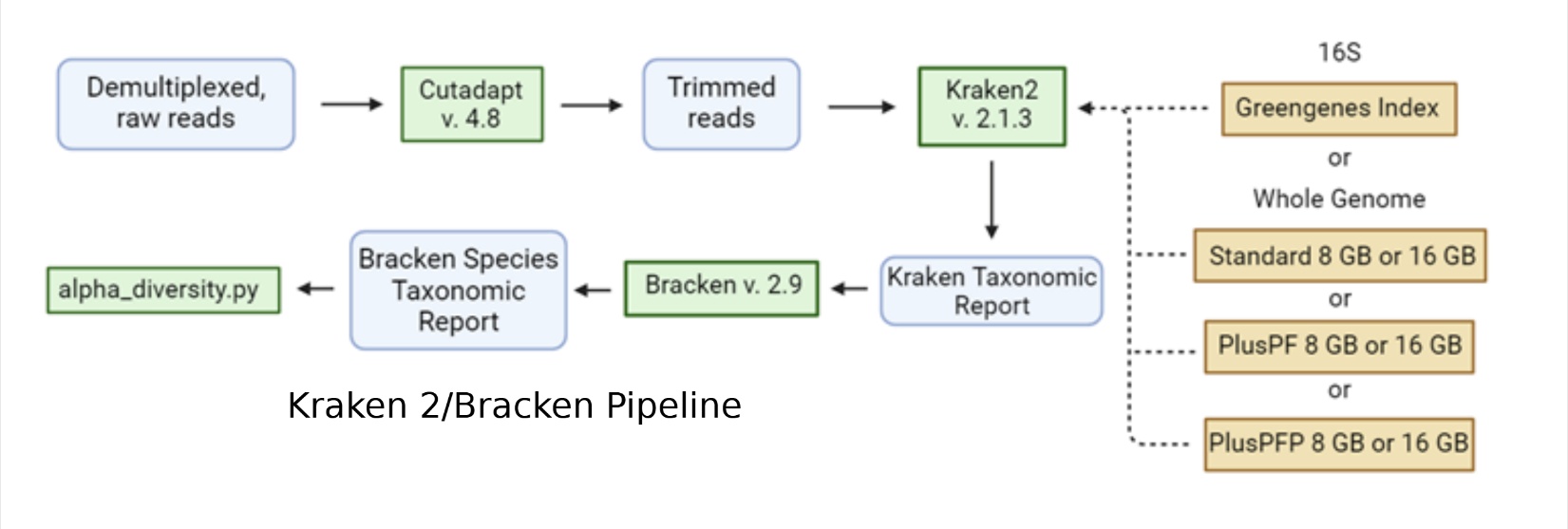


Sup. Figure 9 - Schematic of the pipeline used for Kraken 2/Bracken analysis. Figure created using Biorenderer.


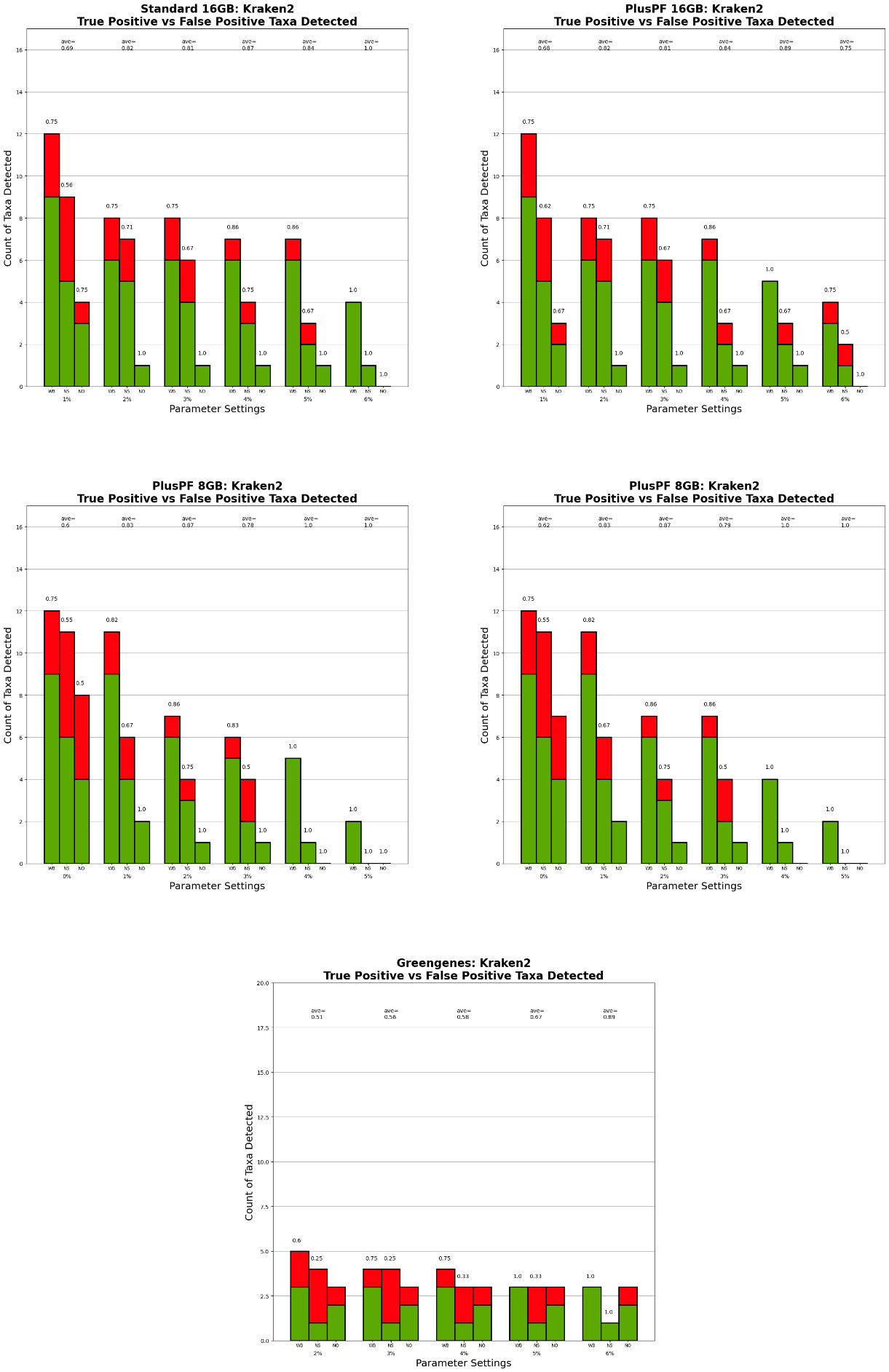


**Sup. Figure 10** – Testing of different Kraken 2 mapping reference databases at varying mapping confidence settings for taxon sensitivity and selectivity using our three simulated read sets. Figure titles indicate the reference database used. Taxa correctly detected are in green, while false positives are in red. Figure created using Matplotlib for Python.


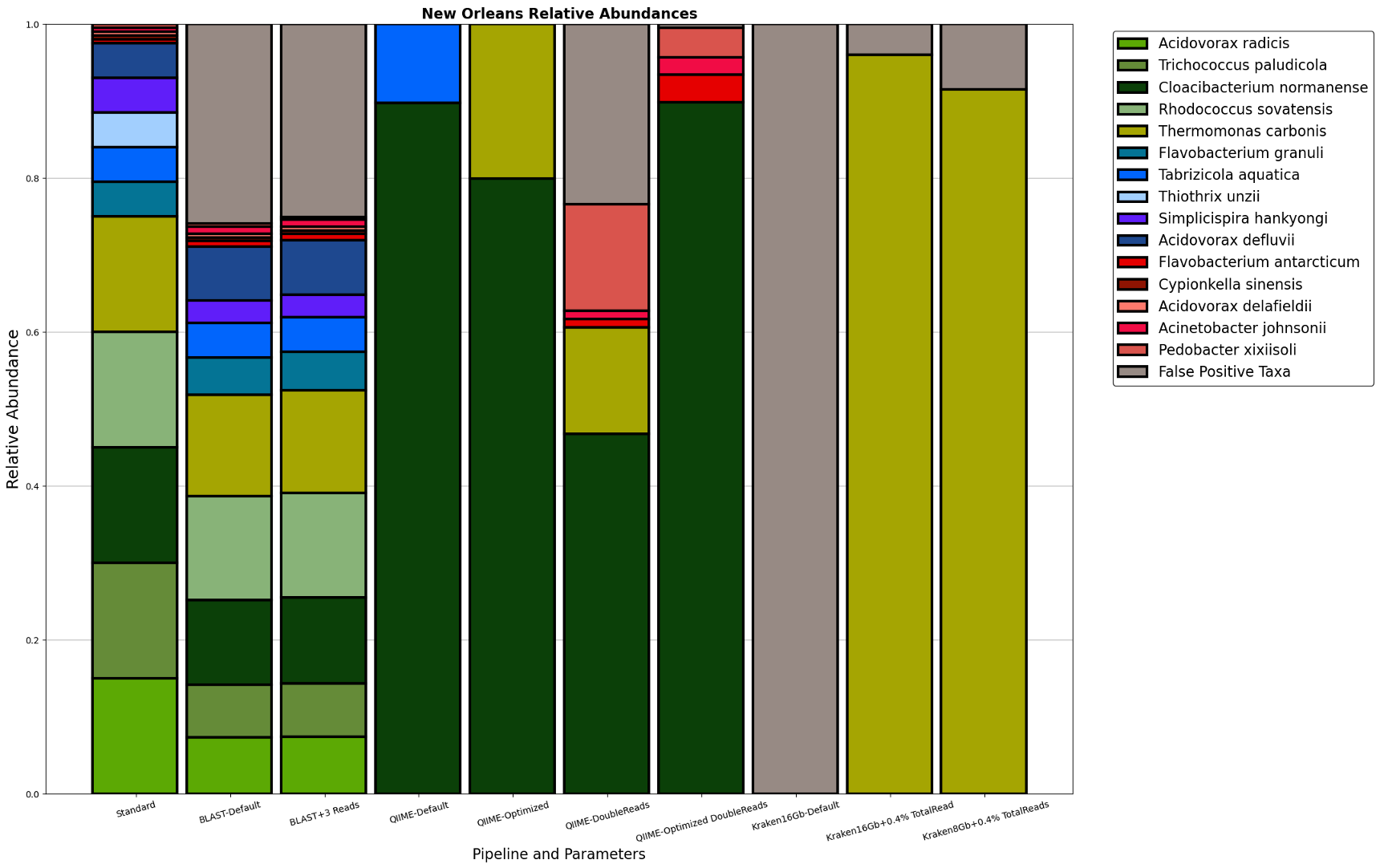


**Sup. Figure 16** – The relative abundance of each taxon present in each simulated read set for the West Bank, the North Shore and New Orleans. Main taxa, comprising an abundance of 15% each, are shown in shades of green, while mid taxa, comprising 4.5% each, are in shades of blue. Finally, rare taxa, making up only 0.5% each, are shown in shades of red. Grey indicates the relative abundance assigned to false-positive taxa. Figure created using Matplotlib for Python.


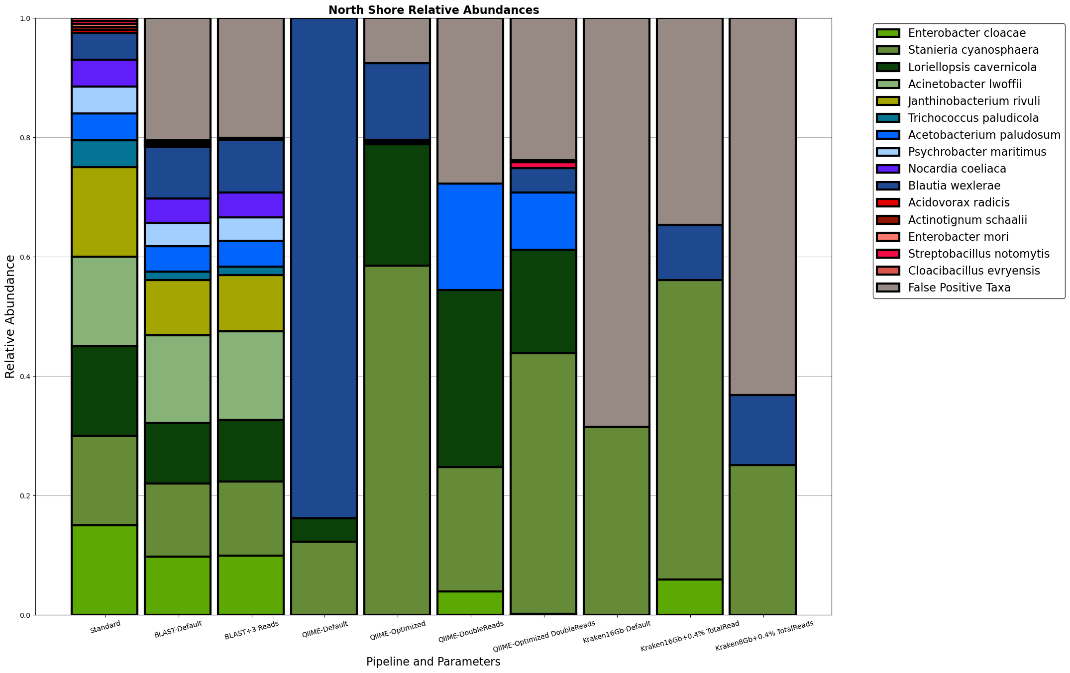


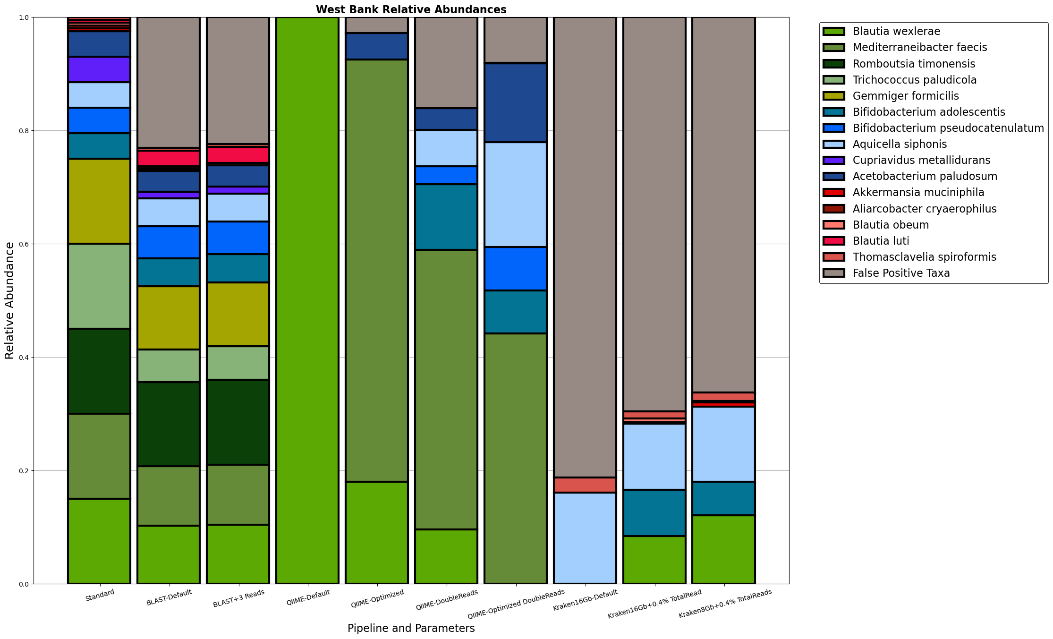


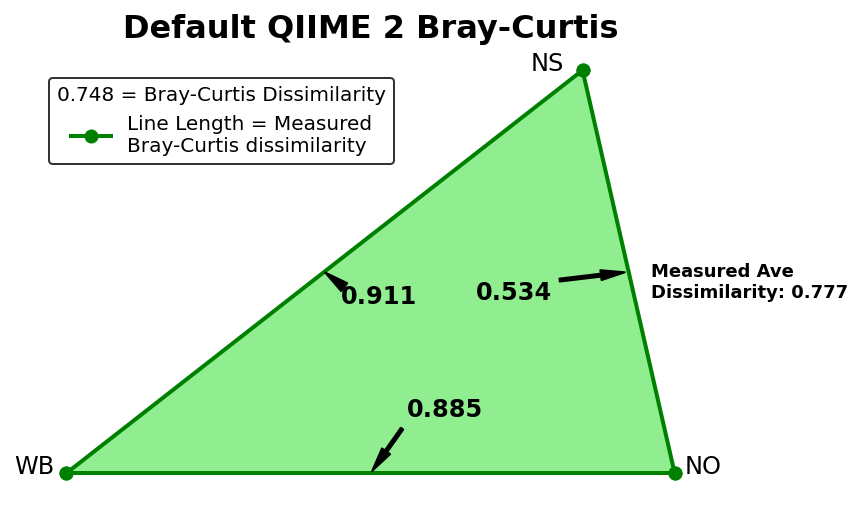


Supplementary Figure 17 – Beta diversity measured by the other pipelines for the three real read sampling locations. The length of each side corresponds to the average Bray-Curtis dissimilarity detected between the two read sets. Average dissimilarities are annotated to the right below the legend.


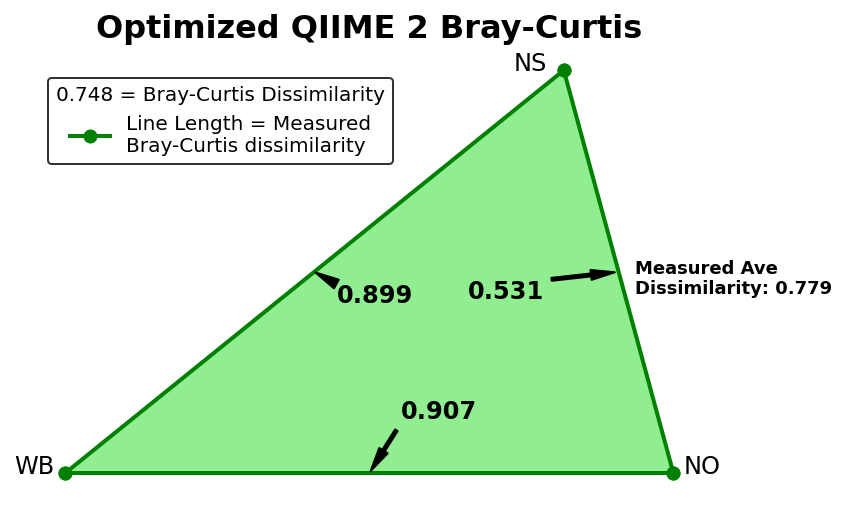

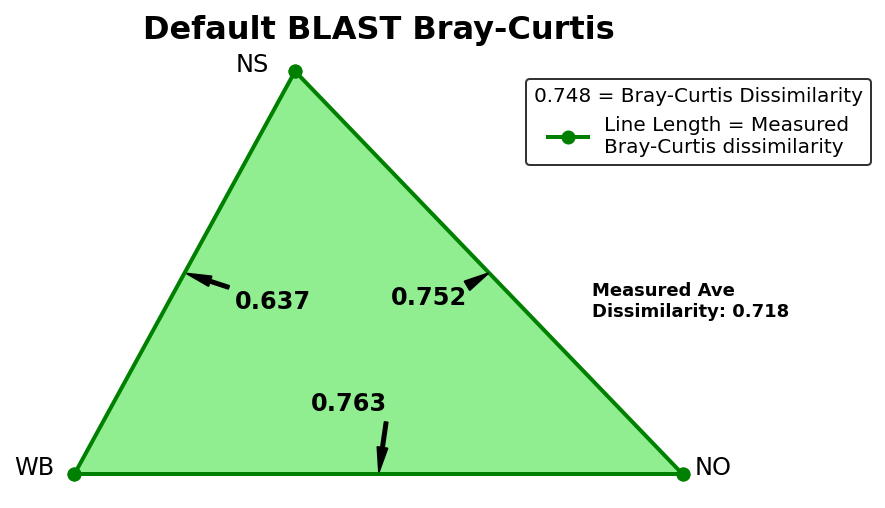

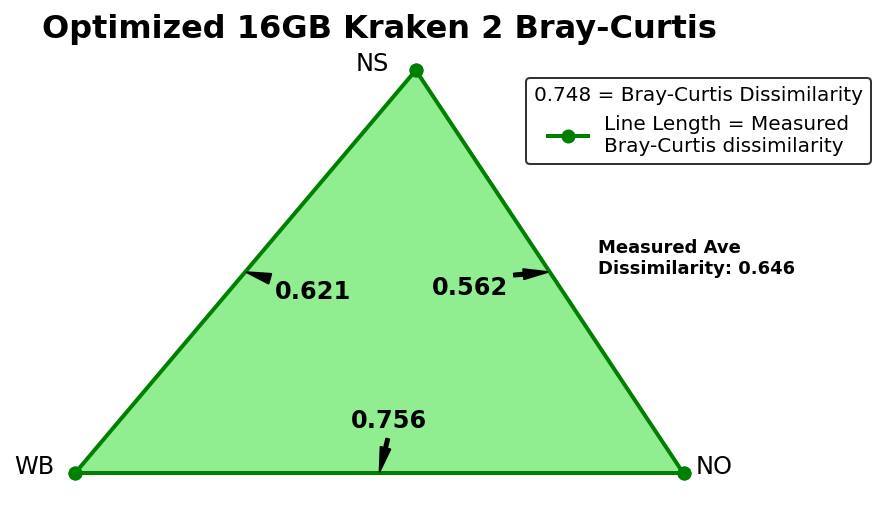

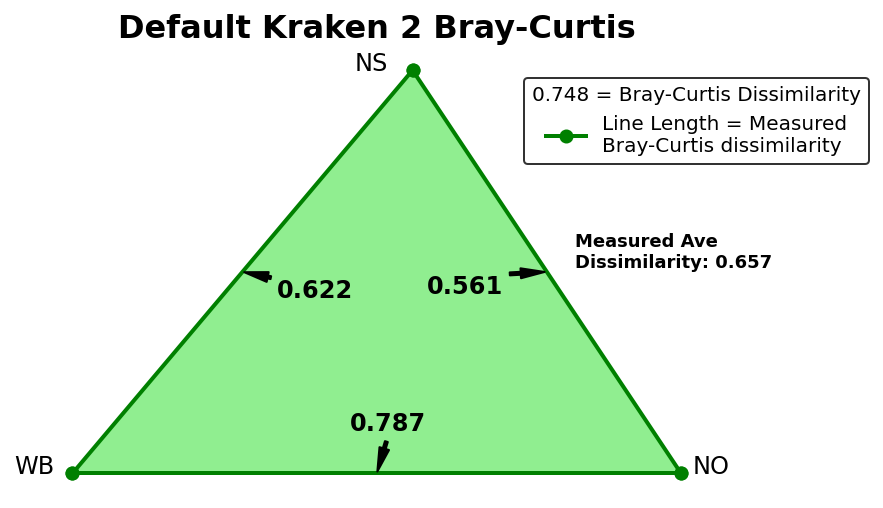

Supplementary Table 1: Split box comparing both simulated and real read results of each taxonomic pipeline. Simulated read results are out of their known true value, while the true values for real reads are unknown. For BLAST Subsampling, Optimized A is with low-read taxa removed. For Kraken 2/Bracken, Optimized A is optimized parameters with the 16GB Standard reference database, while Optimized B is the same with the 8GB Standard reference database. For QIIME 2/DADA 2, Optimized A is the optimized pipeline using single-input reads, while Optimized B is the same for double input reads.
