# Supplementary material for: Flush With Data (or) Optimizing and Validating the Efficacy of Free and Computationally Simple 16S Metabarcoding Approaches for Use in Wastewater Surveillance
Source: Environ Microbiol. 2026 Apr 30;28:e70276. doi: 10.1111/1462-2920.70276 (PMC13130369; doi:10.1111/1462-2920.70276)
Supplement: Supplementary file 1 — Figure S1: Map of the Greater New Orleans, LA area, the three WWTPs that were sampled and their approximate catchment areas: Mandeville (North Shore), New Orleans East Bank (NOLA), and Belle Chasse (West Bank). Image created with ArcGIS. Figure S2: The 16S Amplicon Complex using Bakt_341F and Bakt_805R primers to amplify the V3‐V4 region of the 16S rRNA gene of B. adolescentis genome. Figure created with Biorenderer. Figure S3: Alpha rarefaction curves of OTU counts for each pipeline to determine if adequate sequencing depth was achieved with the Illumina V3 and V2 kits. Plateau regions indicate sample sizes of maximum OTU detection. Figures created with vegan for R. Figure S4: Diversity measures used. For alpha diversity: Richness was the number of taxa or OTUs detected, Chao1 was used to estimate the true richness of each sample, Shannon Entropy was used as a measure of diversity (richness and evenness), and Pielou's Evenness as a measure of community evenness. For beta diversity, Bray–Curtis Dissimilarity was used for the differences in two community structures. Singletons/doubletons refer to taxa represented by a single read or two reads, respectively. Figure created using LaTeX. Figure S5: Species present in each our three simulated wastewater 16S read sets: West Bank, North Shore, and New Orleans. Main taxa (15% relative abundance ea.) are shown in bold italics, Mid Taxa (4.5% relative abundance ea.) are shown in underlined italics, and Rare Taxa (0.5% relative abundance ea.) are shown in italics. Figures created with Biorenderer. Figure S6: Violin plot (Box plot in dashed black lines combined with kernel density estimate in colour‐shaded regions). Comparison of the distributions of simulated reads per taxon mapped either correctly (green) or incorrectly (red) for: (A) BLAST Subsampling, (B) Baseline Kraken 2/Bracken 16 GB, (C) Loosened Kraken 2/Bracken 16GB, (D) Loosened Kraken 2/Bracken 8 GB. Figure created with Seaborn for Python. Figure S7: Schematic of th [file EMI-28-e70276-s001.zip › emi70276-sup-0002-Supinfo1.docx]

**Supplementary Scoring Methods**

Alpha and Beta Diversity Calculations


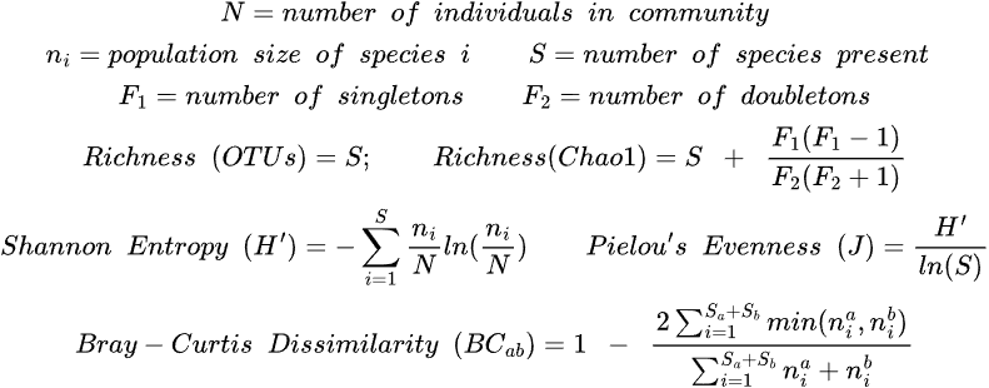
All alpha diversity measures were calculated using the Diversity suite of functions within the Scikit-Bio Python package (Scikit-bio, 2025). **(see Sup. Figure 4)** Sample richness was calculated both using simple Observed Taxonomic Unit (OTU) counts (S) as well as the bias-corrected Chao1 Richness estimator to account for missed rare taxa. Community evenness was measured using Pielou’s Evenness Index (J). Overall diversity was measured using Shannon Entropy (H’). The Bray-Curtis dissimilarity formula was used to score the beta diversity between samples from different wastewater treatment plants (WWTPs).

**Sup. Figure 4** – Diversity measures used. For alpha diversity: Richness was the number of taxa or OTUs detected, Chao1 was used to estimate the true richness of each sample, Shannon Entropy was used as a measure of diversity (richness and evenness), and Pielou’s Evenness as a measure of community evenness. For beta diversity, Bray-Curtis Dissimilarity was used for the differences in two community structures. Singletons/doubletons refer to taxa represented by a single read or two reads, respectively. Figure created using LaTeX.

Significance Testing

The Pearson correlation coefficient was calculated by using the corr() function in the Pandas Python package (McKinney, 2010). Linear regressions were conducted using the OLS() function in the statsmodels.api Python package (API Reference, n.d.) Student’s T-test was conducted using ttest_1samp.() from the SciPi Python package (Virtanen, 2020).

CAMISIM Sensitivity and Selectivity Calculations


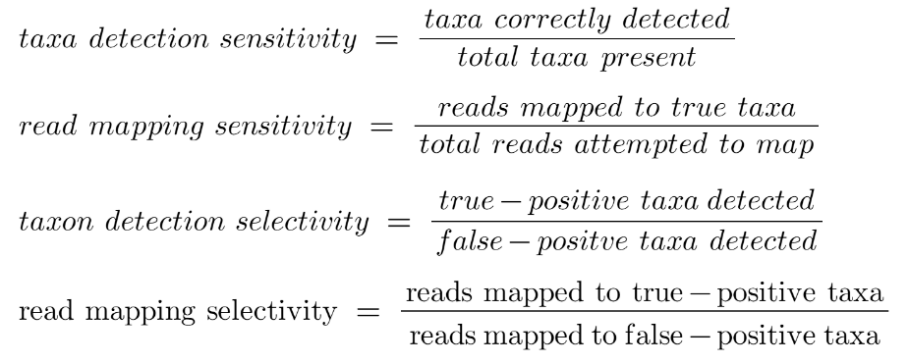
**Taxa Sensitivity** was calculated as the quotient of the total number of simulated taxa correctly detected by the pipeline divided by the total number of simulated taxa truly present in the sample, which for this study was fifteen taxa per simulated sample. **Read mapping sensitivity** was calculated similarly as the sum of all reads the pipeline mapped to simulated taxa truly present in the sample divided by the total number of simulated reads available to be mapped. **(see Sup. Figure 11)**

**Sup. Figure 11** – Formulas used to calculate the taxa/read mapping sensitivity and selectivity of the various pipelines and parameters with CAMISIM simulated read sets. Figure created using LaTex

**Taxon Selectivity** was the quotient of the number of simulated taxa correctly detected divided by the total number of taxa detected. **Read mapping selectivity** was, likewise, the ratio of the number of reads mapped to those same true taxa and those mapped to all taxa.

Overall Species Surveillance Score Calculation

**Sup. Figure 12** – Selection formula for overall read mapping accuracy of our analysis pipelines. Figure created using LaTeX.


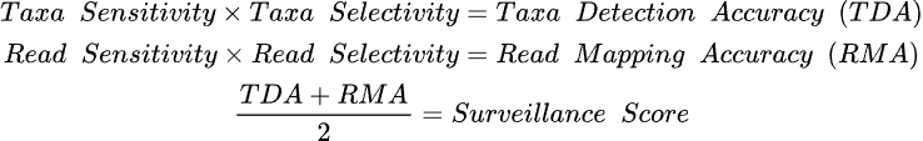
The overall pipeline surveillance score is intended to give an empirical and objective score ranging from zero to one that indicates the general efficacy of the pipeline for the purposes of species-level taxa surveillance. Two main factors were included in the score: the ability of the pipeline to completely and accurately report the taxa present in the sample and the ability of the pipeline to completely and accurately map reads to those taxa. The score was calculated such that the score and the efficacy of the pipeline vary proportionally. **(see Sup. Figure 12)**

To this end, the score is the mean of two values: the Taxa Detection Accuracy (TDA), calculated as the multiple of the Taxa Sensitivity and the Taxa Selectivity scores, (see definitions, above) and the Read Mapping Accuracy (RMA), which is the multiple of the Read Sensitivity and the Read Selectivity (definitions also above).


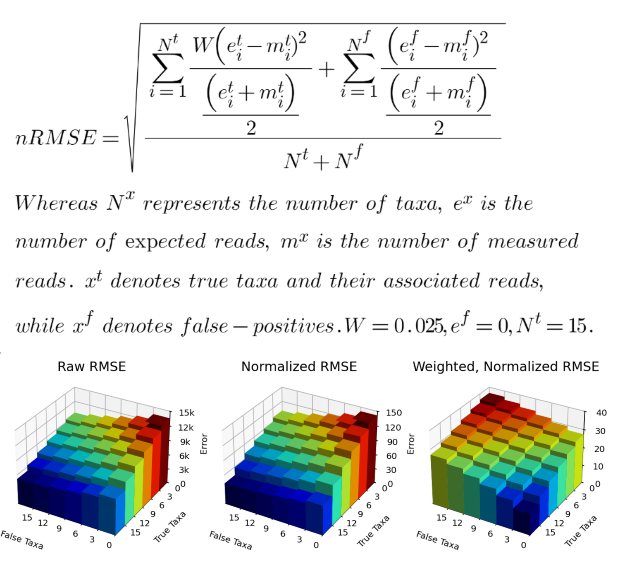
Multiples were used instead of sums to calculate the TDA and RMA too address two extreme cases: a pipeline that was so conservative that it only ever detects one or two taxon per sample but both were correct, and a pipeline so liberal it would identify all present taxa correctly but with so many false-negative taxa that differentiating the two groups would be impractical, if not impossible. If sums were used, either one of these theoretical pipelines would receive a total score above 0.5 over (0,1) as they would have perfect selectivity or sensitivity, respectively, but both pipelines would not have any real power for determining the microbial community present in an unknown sample. It should be noted that in the three pipelines here under study, both extremes were seen when using default or baseline parameters. By using multiples, the TDA and RMA for such cases would be low enough to reflect the true power of the pipeline.

Sup. Figure 14 - Modeling of the standard, read-normalized, and weighted and normalized Sum of Absolute Errors equations when used to quantify read mapping error for various pipelines on CAMISIM simulated read sets. The number of true and false-positive taxa was tested the same as with RMSE in figure 11. Figure created using LaTeX and Matplotlib for Python.

CAMISIM Read Mapping Error Calculations

Read mapping error formulas were modeled using sets of 150,000 theoretical reads, with each “main taxa” true positive having 10,000 out of its 22,500 possible reads mapped to it and each “main taxa” false negative species having zero reads mapped to it. Similarly, each “mid taxa” detected received 4,500 out of its 6,750 possible reads, and each “rare taxa” detected had 350 out of its 750 reads mapped. Each false positive taxon, errantly detected, was assigned 20 reads. These numbers were typical of the values observed during analysis of actual samples.


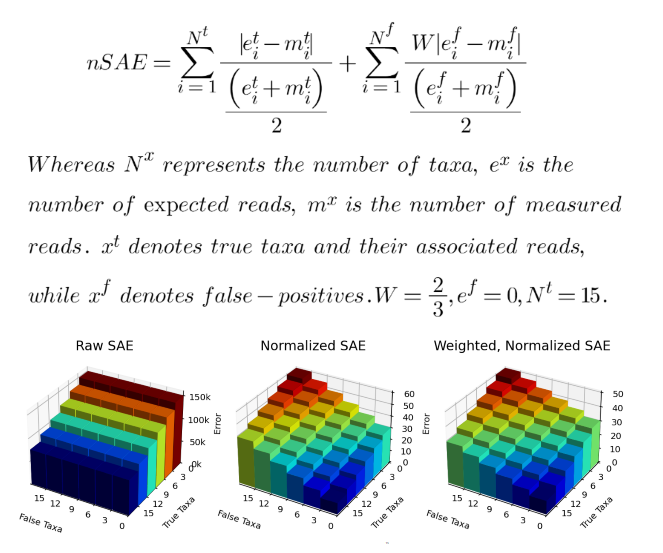
The measurement power of both Root Mean Squared Error (RMSE) and Sum of Absolute Errors (SAE) formulas were modeled by varying the number of true and false-positive taxa detected. The correlation and significance between the proportion of reads correctly or incorrectly detected were then compared to the total mapping error measurement using Pearson correlation and linear regression. True positive taxa were increased from zero to fifteen in intervals of three so as to add one detected taxa to the main, mid and rare taxa groups in each successive interval to control for the varied impact taxa from each group has on the final read mapping error, while false-positive taxa were also increased over a range or zero to fifteen in increments of threes so as to match the incremental increases of the true taxa. **(see Sup. Figures 13 & 14)**

**Sup. Figure 13** – Modeling of the standard, read-normalized, and weighted and normalized Root Mean Square Error equations when used to quantify read mapping error for various pipelines on CAMISIM simulated read sets. Each equation was tested using between zero to fifteen different true and false positive taxa detected, increasing by intervals of three. For true taxa detected, each increase of three involved the addition of one main, one mid and one rare taxon detected. Figure created using LaTeX and Matplotlib for Python.


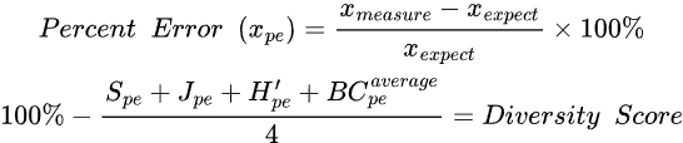
 (nRMSE) Two different calculation methods were used to evaluate the analysis pipelines’ read mapping error rate. The standard Root Mean Square Error (RMSE) calculation was augmented by dividing each term by the arithmetic mean of the expected and measured reads to normalize the error calculation regarding the total number of reads in the set (nRMSE), as has been done elsewhere (Odom et al, 2023). While RMSE and nRMSE are the read mapping error calculations typically methods typically seen; we noticed a potential setback that RMSE-based formulas have in calculating mapping error: since the total read-mapping error sum is divided by the total number of taxa detected, and false-positive taxa generally have far fewer reads errantly assigned to them than true taxa have reads missed, a pipeline generating many false-positive taxa will have a speciously suppressed total error value due to its disproportionately enlarged denominator. **(see Sup. Figure 13)** To attempt to remedy this, the errors generated from false-negative reads were weighted by multiplying their sum by 0.025, equalizing the impact of type I and type II errors on the final score. This resulted in an error rating that more closely reflected the percentage of true-to-false-positive taxa detected and the ratio of reads mapped to them.

Sup. Figure 15 – Diversity score is calculated with Richness, Pielou’s Evenness, Shannon Entropy, and the average value of Bray-Curtis Dissimilarity between the three test sites. The percent error was found for each by comparing the measured and expected value. The mean of these four percent error measures is then subtracted from 100% so the score will vary proportionally with the usefulness of the pipeline for making accurate diversity estimates. Figure created using LaTeX.

(nSAE) A different mapping error strategy – one that does not involve dividing the error by the total number of taxa detected – is Sum of Absolute Errors (SAE). The nSAE calculation was normalized to the total read number in the same fashion as the nRMSE, **(see Sup. Figure 14)** with an additional weight applied by multiplying the sum of the error generated from reads mapped to false-positive taxa by 0.667. This weight equalized the influence both false-positives and false-negatives reads have on the final error measurement.

Overall Diversity Score Calculation

The overall pipeline diversity score, like the surveillance score (see definition above), is intended to give an objective score ranging from zero to one that varies proportionally to the general efficacy of the pipeline for bacterial population diversity measures. **(see Sup. Figure 15)** The score is the complement of the mean of four percent error values. The first is on the pipeline’s ability to correctly measure species richness, here indicated by the percent error in the pipeline’s determination of OTUs present in the sample. The second value is the measure of the pipeline’s ability to correctly assess sample evenness using the percent error of Pielou’s Evenness Index. The third is the evaluation of the pipeline’s ability to gauge overall alpha diversity, here using the percent error of Shannon Entropy. The final value is percent error of Bray-Curtis Dissimilarity measurements, to represent the pipeline’s ability to gauge beta diversity values.

**References**

1. API Reference — statsmodels. (n.d.). Www.statsmodels.org. https://www.statsmodels.org/stable/api.html
2. McKinney, W. (2010). Data Structures for Statistical Computing in Python. Proceedings of the 9th Python in Science Conference, 445. https://doi.org/10.25080/majora-92bf1922-00a
3. Odom, A. R., Faits, T., Castro-Nallar, E., Crandall, K. A., & Johnson, W. (2023). Metagenomic profiling pipelines improve taxonomic classification for 16S amplicon sequencing data. Scientific Reports, 13(1). https://doi.org/10.1038/s41598-023-40799-x
4. Virtanen, P., Gommers, R., Oliphant, T. E., Haberland, M., Reddy, T., Cournapeau, D., Burovski, E., Peterson, P., Weckesser, W., Bright, J., van der Walt, S. J., Brett, M., Wilson, J., Millman, K. J., Mayorov, N., Nelson, A. R. J., Jones, E., Kern, R., Larson, E., & Carey, C. J. (2020). SciPy 1.0: fundamental algorithms for scientific computing in Python. Nature Methods, 17(3), 261–272.
5. Scikit-bio: Bioinformatics in Python — scikit-bio. (2025). Scikit.bio. <https://scikit.bio/index.html>
